# Comprehensive evaluation of electric vehicle charging network under the coupling of traffic network and power grid

**DOI:** 10.1371/journal.pone.0275231

**Published:** 2022-09-23

**Authors:** Lixun He, Jun He, Liwen Zhu, Wentao Huang, Yanyang Wang, Hua Yu

**Affiliations:** 1 Hubei Collaborative Innovation Center for High-Efficiency Utilization of Solar Energy, Hubei University of Technology, Wuhan, China; 2 Yichang Power Supply Company, State Grid Hubei Electric Power Co., Ltd., Yichang, China; Beijing Institute of Technology, CHINA

## Abstract

With the rapid development of electric vehicle (EV) technology under the background of double carbon, a reasonable evaluation of the network planning of EV charging stations is conducive to the long-term stable development of the EV industry. Therefore, this paper proposes a comprehensive evaluation system and method for EV charging networks under the traffic network and power grid coupling. First, an EV travel model is developed based on the travel probability matrix to analyze the spatial and temporal characteristics of EVs. Secondly, the coupling relationship among users, charging network, road network, and the power grid is analyzed, four criteria of user feedback, charging network operation, road network operation impact, and power grid operation impact are proposed, and corresponding evaluation indexes are constructed under each criterion to form a comprehensive evaluation index system. Furthermore, the Analytic Hierarchy Process (AHP) and entropy weighting method are used to assign weights to the index layer and the criterion layer respectively, and the improved TOPSIS evaluation method is used to quantitatively analyze the impact of charging networks on different subjects for comprehensive evaluation. Finally, through the comprehensive evaluation of different charging network planning schemes, the effectiveness of the comprehensive evaluation method and model proposed in this paper is verified, which can provide a reference basis for the planning and improvement of EV charging station networks.

## 1. Introduction

In the face of increasing global energy shortage and environmental pollution, governments around the world are vigorously promoting EVs, which have the potential to save fossil fuels, reduce carbon emissions and increase the penetration of renewable energy [1, 2]. The number of EVs in China reaches 6.4 million in 2021, accounting for 81.63% of the total number of new energy vehicles. With the large-scale EV access, a perfect charging station service network is the foundation to guarantee the stable development of the EV industry [3, 4]. However, due to the randomness of the spatial and temporal distribution of EV charging load, the subjectivity of users’ charging behavior, the unreasonable construction location of charging stations and the configuration of charging facilities in the stations, etc., it leads to the problems of high grid carrying pressure, long waiting time of users, road blockage and low utilization rate of charging facilities. To guarantee the stable development of EVs and reasonably evaluate the charging network planning scheme, a comprehensive evaluation system of EV charging network considering grid, road network, charging network, and user side multi-dimension is proposed.

At present, the impact assessment of large-scale EV access to the distribution network is one of the hot issues of domestic and international research. Large-scale EV access will bring major challenges to the safety, stability, and economic operation of the distribution network, causing line overload, voltage collapse, increased network loss, transformer overload, three-phase asymmetry, and other problems [5]. Researchers have focused on the ability of distribution grids to accommodate EVs in terms of economy, reliability, safety, coordination, and efficiency. The literature [6] analyzes the voltage stability in a harsh charging environment based on a continuous current model and analyzes the maximum number of EVs that can be connected to the distribution network. The literature [7] evaluated the economic impact of EV charging systems on the distribution network in terms of both charging costs and benefits. The literature [8] evaluates the impact of EV access on network loss, load, and voltage from a spatial and temporal perspective based on an EV charging load prediction model that incorporates information from multiple sources. The literature [9] proposes a road-electric coupling-based EV charging load prediction model for the reliability assessment of EV access to the distribution network.

In addition, most studies have focused on the performance evaluation of charging stations and their supporting facilities. The literature [10] proposes a data-driven battery capacity diagnosis method based on the data, which can be used for the quantitative diagnosis of abnormalities in the charging capacity of different types of EVs. With the development of EV charging technology, some EVs use battery swapping stations (BSS) to replenish their power. In BSS deployment studies, EV driving data are usually used to solve for the optimal layout [11, 12]. In [13], a BSS model combined with deep reinforcement learning is proposed to directly output the optimal real-time charging and discharging power for multiple charging piles, using electricity price and battery demand as inputs. A two-stage optimization with recourse is used in the literature [14] to coordinate the planning and operation of BSS. The literature [15] proposes a BSS optimal operation model to minimize the daily operation cost of the BSS while considering the existing constraints.

However, most EVs still use charging posts to obtain electricity. EV charging posts usually have two charging modes: slow charging and fast charging. In the slow charging mode: the literature [16] used the AHP-entropy weight method evaluation model to construct a charger performance evaluation system with three criteria: general performance, electrical performance, and safety performance. The literature [17] proposed to construct a dynamic evaluation method of charging station service capability with two aspects: service efficiency of charging stations and service perception of users. In fast charging mode: the literature [18] integrates actual traffic data and EV travel patterns to evaluate the instantaneous power demand of fast charging stations based on the evaluation process and the temporal distribution of charging demand. In [19], an advanced measurement system data-driven error state assessment method for metering operation of EV DC charging facilities is proposed. The literature [20] evaluates the operation of fast charging stations based on the operator’s profit and the customer’s waiting time in queues and proposes a pricing strategy to optimize the load and waiting time of fast charging stations. The literature [21] considers the differences between fast charging stations in urban, suburban, and rural areas to analyze the load and customer service quality within fast charging stations.

There are relatively few current studies on the evaluation of charging networks. The literature [22] analyzed the correlated interactions under the coupling of the distribution network, charging network, and road network, and established a comprehensive evaluation index system under the coupling interaction of the three networks. In literature [23], a comprehensive evaluation index system for EV fast-charging network was constructed based on the interaction of four aspects: users, charging network, road network, and distribution network. At present, the AHP-entropy weight method and the fuzzy comprehensive evaluation method are usually used in the comprehensive evaluation of EV charging stations [16, 23, 24]. However, the above methods are highly subjective, which may lead to a lack of objectivity in the evaluation results.

The TOPSIS has been widely used in the field of smart grid and integrated energy systems, and its evaluation results are not affected by the number of indicators [25, 26]. However, few studies have used the TOPSIS approach to evaluate EV charging networks. Although the method has low requirements for raw data and is highly adaptable, the traditional method cannot objectively and effectively reflect the distance between the evaluation scheme and the optimal ideal solution, and other methods need to be used to improve it to achieve the purpose of comprehensive evaluation.

This paper proposes a comprehensive evaluation method for the planning scheme of EV charging station networks under the coupling of the transportation network and power grid. Firstly, a road network model considering speed-flow and a model of the spatial and temporal characteristics of EV travel are used for EV travel simulation. Secondly, according to the road-electricity coupling network relationship, a comprehensive evaluation system of the charging network containing the target layer, criterion layer, and indicator layer is constructed in terms of user feedback, charging network operation, road network operation impact, and power grid operation impact. Then, using AHP and entropy weighting method to assign weights to the criterion layer and indicator layer respectively, based on which an evaluation method combining TOPSIS and grayscale correlation was constructed. Finally, the operational effectiveness of the charging network is quantitatively evaluated by conducting a comprehensive evaluation of the charging network planning scheme. The proposed method can provide a reference for the improvement and refinement of charging network planning.

## 2. Modeling of EV charging load based on origin and destination analysis

### 2.1. Road network model considering speed and flow

#### 2.1.1. Road network topology

**Fig 1:**
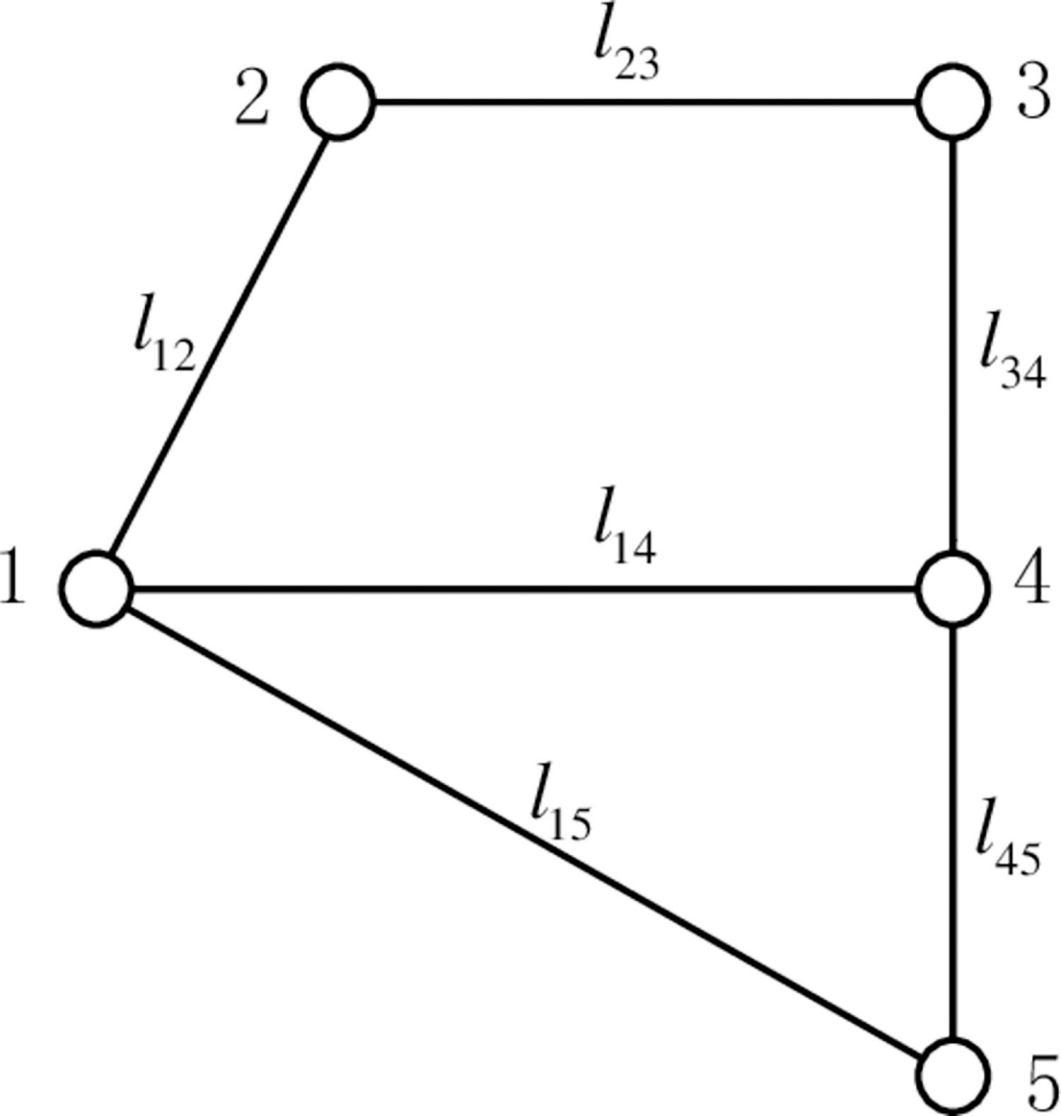
Road network topology.

According to the graph theory, the urban road network is abstracted into a road network topology of nodes and line segments, which is represented by G(V, F). Where V is the set of road intersections in the road network; F is the set of road section lengths between road network nodes. The road network topology matrix D represents the relationship between the nodes in the road network and the length of each road segment. The values of the elements in the topology matrix D are determined by Eq (1), the topology matrix D is shown in Eq (2), and the road network topology is shown in Fig 1.


$$d_{ij}=d_{ji}=\{\begin{array}{cc} l_{ij}, & \left(i,j)\;\mathrm{is}\;\mathrm{the}\;\mathrm{edge}\;\mathrm{in}\;F(G\right)\\ 0, & i=j\\ \inf, & \left(i,j)\;\mathrm{is}\;\mathrm{not}\;\mathrm{the}\;\mathrm{edge}\;\mathrm{in}\;F(G\right) \end{array}$$ (1)

$$D=[\begin{array}{ccccc} 0 & l_{12} & \inf & l_{14} & l_{15}\\ l_{12} & 0 & l_{23} & \inf & \inf\\ \inf & l_{23} & 0 & l_{34} & \inf\\ l_{14} & \inf & l_{34} & 0 & l_{45}\\ l_{15} & \inf & \inf & l_{45} & 0 \end{array}]$$ (2)

#### 2.1.2. Consider the velocity-flow model for real-time traffic flow

In urban road networks, road capacity and traffic flow jointly determine the speed of vehicles [27]. The driving speed of EVs directly affects the power consumption per unit mileage, and also indirectly affects the state of charge (SOC) and charging location. A speed-flow model considering real-time traffic flow is constructed to obtain the real-time traffic speed and flow of each road segment.

$$\left\{ \begin{array}{l} v_{ij,t}=\frac{v_{ij,0}}{1+{\left(C_{ij,t}/C_{ij,0}\right)}^{\beta_{ij}}}\\ \beta_{ij}=\varphi +\gamma {\left(C_{ij,t}/C_{ij,0}\right)}^{3} \end{array}\right.$$ (3)

where *v*_*ij*,*t*_ is the speed of vehicles on the road section *(i*,*j)* at moment *t*, related to the road section flow and road section level; *v*_*ij*,0_is the free travel speed at zero flow for the road section *(i*,*j)* and takes the value of 60km/h; *C*_*ij*,*t*_ is the section flow of section *(i*,*j)* at moment *t*; *C*_*ij*,0_ is the rated capacity of the road section *(i*,*j)*; *φ* and *γ* denote the adaptive coefficients for different road levels; In this paper, the roads are divided into I and II roads, and the values of *φ* and *γ* are taken as 1.726 and 3.15 for I roads and 2.076 and 2.870 for II roads, respectively.

According to the speed-flow model, the passing speed of all road sections at each moment can be calculated, combined with Dijkstra’s shortest path algorithm to plan the EV’s shortest driving path, and then solve for the travel time Δ*T*_*ij*_.

$$\Delta T_{ij}=\sum_{h=1}^{I}\frac{d_{h}}{V_{h,t}}$$ (4)

where *I* is the total number of road sections included in the road network; *d*_*h*_ is the length of section *h*; *V*_*h*,*t*_ is the passing speed of the road section *h* at time *t*.

### 2.2. Analysis of the spatial and temporal characteristics of EV travel

#### 2.2.1. EV travel probability matrix

The Origin-Destination (OD) analysis method is mainly used to describe the traffic distribution in the road network, and its core is the OD travel probability matrix, which can describe the travel trajectory of EVs. Based on the traffic flow at each starting and ending point in the road network, the OD matrix is formed. The rows of the matrix represent the traffic generation at each starting point and the columns represent the traffic attraction at each ending point. The OD travel probability matrix can be described by Eq (5).

$$p_{ij}^{t,t+1}=b_{ij}^{t,t+1}/\sum_{j=1}^{m_{r}}b_{ij}^{t,t+1},\;1\leq i,j\leq m_{r}$$ (5)

where $p_{ij}^{t,t+1}$ is the probability of travel demand for EVs traveling from node *i* to node *j* in the traffic road network from time period *t* to *t*+1; $b_{ij}^{t,t+1}$ is the number of EVs driving from node *i* to node *j* in time period *t* to *t*+1; *m*_*r*_ is the number of road network nodes.

#### 2.2.2. EV power consumption model considering temperature and speed

In most EV charging load prediction studies, the EV power consumption is usually set to a constant value (ignoring driver’s behavior, external factors, etc.) and the power consumption is considered to be linearly related to the driving distance [28, 29]. During actual driving, the EV owner’s driving behavior will have a direct impact on the vehicle’s battery status, such as by the ambient temperature, driving speed, and other factors. The environment temperature affects the performance of the battery, which in turn leads to changes in vehicle energy consumption. Changes in environmental temperature also directly lead to additional power consumption from air conditioning operations. When traffic is congested, the decrease in driving speed also leads to an increase in EV power consumption [30]. Therefore, a real-time power consumption model of EV considering temperature and speed is constructed in this paper as follows.

$$E_{temp}=\{\begin{array}{l} P_{cool}\frac{\Delta l}{v_{ij,t}}\;T_{k}>T_{k,\max}\\ P_{heat}\frac{\Delta l}{v_{ij,t}}\;T_{k}>T_{k,\min} \end{array}$$ (6)

$$E_{speed}=0.21-0.001v_{ij,t}+\frac{1.531}{v_{ij,t}}$$ (7)

$$E_{Tk}=E_{temp}+E_{speed}$$ (8)

where *E*_*temp*_ denotes the electricity consumed by the EV traveling *Δl* km at speed *v*_*ij*,*t*_ with the air conditioner when the environmental temperature is *T*_*k*_; *T*_*k*,*max*_ is the lower limit of air conditioning cooling temperature; *T*_*k*,*min*_ is the lower limit of air conditioning cooling temperature; *P*_*cool*_ and *P*_*heat*_ are the cooling and heating power of the air conditioner, respectively. *E*_*speed*_ indicates the real-time power consumption per unit kilometer of the EV at different driving speeds; *E*_*Tk*_ indicates the real-time power consumption per unit kilometer when the EV is driving at speed *v*_*ij*,*t*_ at environmental temperature *T*_*k*_.

#### 2.2.3. EV charging time

The charging demand of EV is determined by the State of Charge (SOC), and the initial SOC is assumed to obey the normal distribution *N*_*SOC*_ (0.6,0.1^2^). To evaluate the capability of the charging network more effectively, electric cabs were selected as the target of the study, and the charging behavior was in fast charging mode. The charging time *T*_*c*_ can be expressed as follows:

$$T_{c}=\frac{(SOC_{f}-SOC_{t})Cap_{r}}{\eta_{c}P_{c}}$$ (9)

where *SOC*_*f*_ is the state of charge at which the EV completes charging which obeys a normal distribution *N*(0.85,0.3) [31]; *SOC*_*t*_ is the state of charge of the EV entering the charging station at moment *t*; *Cap*_*r*_ is the rated battery capacity of the EV; *η*_*c*_ is the charging efficiency of the charging post; *P*_*c*_ is the rated charging power of the EV.

To obtain EV charging load more accurately, this paper classifies charging users into demand type and random type. If the current remaining power cannot complete the next leg of the journey, the EV must be recharged at this time, and such cases belong to demand-type users. When users have relatively sufficient power, users who generate charging behavior due to the psychology of power anxiety belong to random type users. The specific classification is as follows.

$$\left\{ \begin{array}{l} \mathrm{Demand}\;\mathrm{type}\;SOC_{t,1}\leq 20\%\\ \mathrm{Random}\;\mathrm{type}\;SOC_{t,1}>20\% \end{array}\right.$$ (10)

where *SOC*_*t*,*1*_ is the SOC of the EV at the end of the next leg of the trip. In this paper, we refer to the fuzzy inference-based charging decision model for random-type users proposed in literature [32] to calculate the charging probability of random-type users.

### 2.3. Charging station queuing system

The average waiting time is closely related to the queuing format, and the charging station queuing system is set to a shared queue mode. The shared queue in the charging process indicates that the EV queuing method is a single queue and the charging posts are in a parallel service state. The service intensity of the shared queue is expressed as follows:

$$\rho_{s}=\frac{\lambda_{a}}{N_{i}\mu_{t}}$$ (11)

The wait time of the shared queue is expressed as follows.

$$\bar{T_{w}}=\frac{\rho_{s}}{\lambda_{a}}\frac{{\left(N_{i}\rho_{s}\right)}^{N_{i}}}{N_{i}!}\frac{{\tilde{q}}_{s0}}{{\left(1-\rho_{s}\right)}^{2}}$$ (12)

$${\tilde{q}}_{s0}={\left[\sum_{k=0}^{N_{i}-1}\frac{{\left(N_{i}\rho_{s}\right)}^{k}}{k!}+\frac{{\left(N_{i}\rho_{s}\right)}^{N_{i}}}{N_{i}!}\frac{1}{1-\rho_{s}}\right]}^{-1}$$ (13)

where *λ*_*a*_ is the arrival rate; *N*_*i*_ is the number of chargers; *μ*_*t*_ is the service time, when the service intensity is less than 1, the queuing system can run stably [33]; ${\tilde{q}}_{s0}$ is the probability that the shared queue has no queueing circumstances.

## 3. Comprehensive evaluation system of charging network under transportation network-grid coupling

### 3.1. Analysis of road-electric coupling network relationship

**Fig 2:**
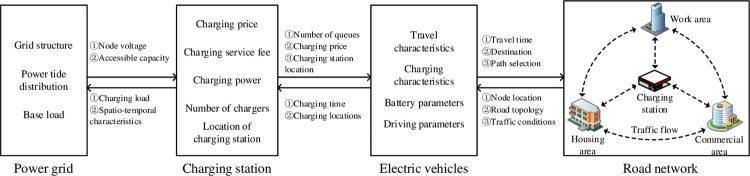
Road-electric coupling network interaction architecture.

EVs and charging stations act as the media of multi-source information transmission in road-electric coupling networks. The access of large-scale EVs makes the power grid and road network gradually become a coupling network that influences and supports each other. EV charging demand is influenced by the subjective willingness of users, road network structure, and vehicle flow. When EVs are charged at charging stations, EVs are connected to the grid as charging loads, and the grid tide distribution will change. The road network and power grid coupling network architecture is shown in Fig 2.

In the transportation network and power grid coupling network, the specific interaction process of multi-source information is as follows:

1. The node voltage and access capacity of the power grid affect the spatial and temporal distribution of the charging station. Meanwhile, the charging station is used as a special load connected to the distribution network, and its charging load affects the operation of the distribution network;
2. The charging station provides charging services for EVs according to the charging demand of users. At the same time, the charging station receives the accessible capacity, node voltage and time-sharing electricity price information from the distribution network and feeds back the spatial and temporal distribution characteristics of the charging load to the distribution network;
3. EV selects the best travel route according to the information feedback from the road network, and the traffic situation in the road network also changes. At the same time, EV owners make charging decisions according to the operation status and charging the price of the charging station;
4. The road network provides users with real-time road congestion and traffic flow, which in turn affects the travel characteristics of users. Simultaneously, the travel path of EVs also affects traffic conditions.

### 3.2. Selection of evaluation indicators

The charging behavior and travel routes of EVs are stochastic, and EVs as a medium will facilitate deep coupling interaction of road networks, power grids, and charging networks. The charging network is powered by the power grid. EV, as a mobile energy storage device, realizes the information interaction between the power grid and the charging network. At the same time, when EVs are connected to the charging network, the charging network provides them with charging guidance and parking functions, as well as being an important part of the modern transportation road network.

**Fig 3:**
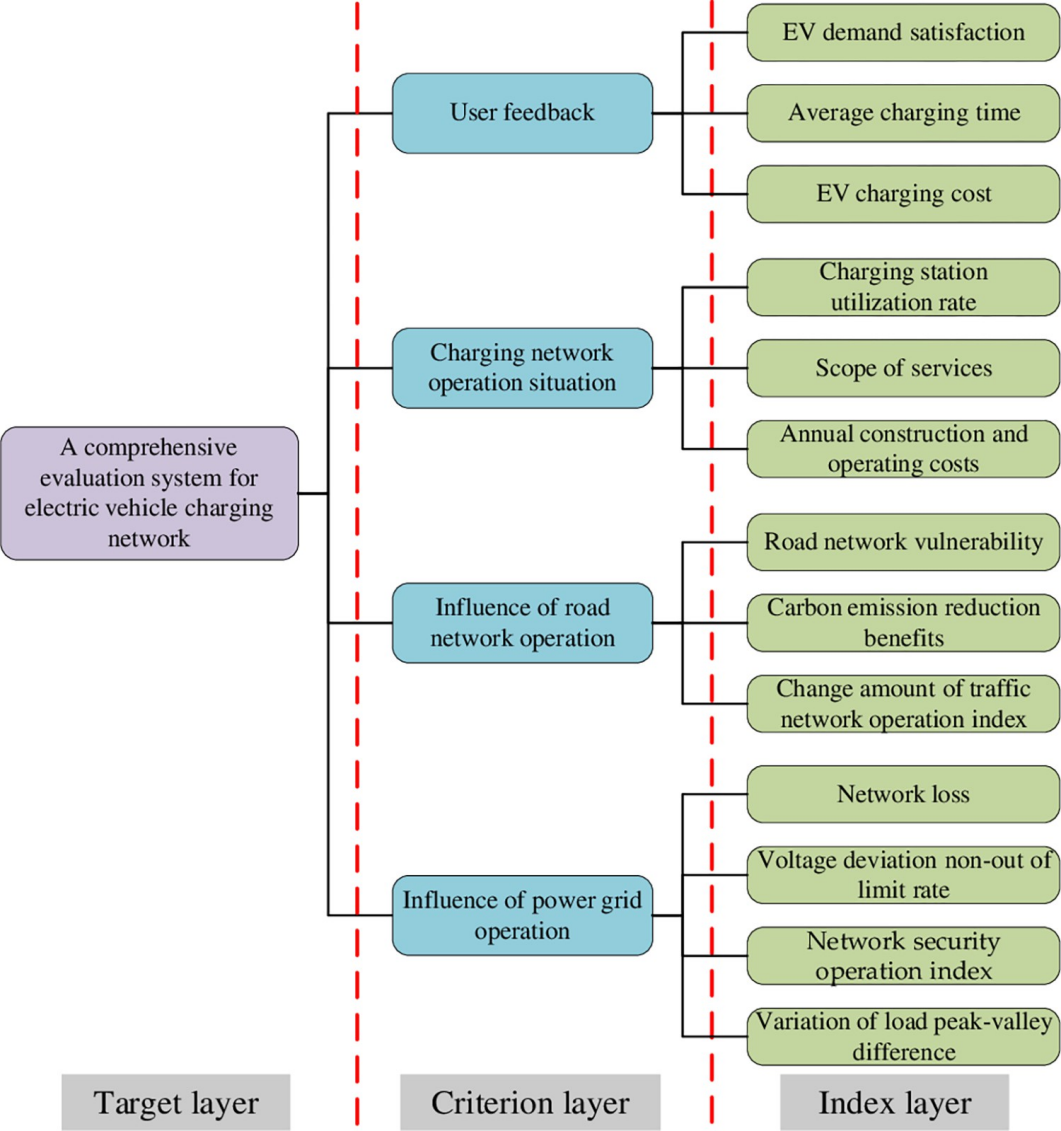
Comprehensive evaluation index system of charging network. 1. User feedback: From the user side, the user’s charging experience is used to measure the service quality of the charging network, to construct EV demand satisfaction and average charging time; From the economic point of view, the charging cost index for vehicle owners is constructed. 2. Charging network operation: From the charging station operation and economic perspective, station utilization rate, service scope, and annual construction and operation cost indicators are constructed, which can visually reflect the service capability of the charging network. 3. Road network operation impact: Since EV access to the charging network will lead to changes in the flow and congestion of each road in the road network. Therefore, indicators such as road network vulnerability, carbon emission reduction benefits, and the amount of change in road network operation index are constructed. 4. Influence of power grid operation: From the perspective of safe operation of the distribution network, EV charging load is characterized by flexibility and randomness and exists in the power grid as a special load. The indexes of network loss, voltage deviation non-out of limit rate, network security operation index, and variation of load peak-valley difference are constructed.

According to the characteristics of users, grid, charging network, and road network, and considering the principles of economy, safety, reliability, and environmental protection to construct the comprehensive evaluation index of charging network under road-electric coupling network from multi-dimension, the specific process is as follows. Based on this, a comprehensive evaluation system of the charging network containing the target layer, criterion layer, and index layer is constructed, as shown in Fig 3.

## 4. Model for calculating comprehensive evaluation index of charging network

### 4.1. User feedback evaluation indicators

EV demand satisfaction, average charging time, EV charging cost, and other indicators were constructed to comprehensively evaluate the charging network service from the perspective of user evaluation and economy.

#### 4.1.1 EV demand satisfaction

EV demand satisfaction *S*_*EV*_ directly reflects the advantages and disadvantages of charging network services through the user side, while *S*_*EV*_ is determined by both user relevance and the number of charging stations [34].

$$S_{gld}=\frac{\sum_{m=1}^{M}\left(\frac{\sum_{h_{m}=1}^{H_{m}}d_{hm}}{d_{Total}}+\frac{\sum_{t=1}^{T}\sum_{n_{m}=1}^{N_{m}}n_{m,t}}{TN_{EV}}\right)}{M}$$ (14)

where *S*_*gld*_ is the influence of correlation degree on EV demand satisfaction; *M* is the number of charging stations; *d*_*hm*_ is the length of the section *h*_*m*_ connected to the charging station *m*; *h*_*m*_ and *H*_*m*_ are respectively the road section *h*_*m*_ and the total number of road sections connected to the *m*th charging station, *h*_*m*_∈*H*_*m*_; *d*_*Total*_ is the total length of all road sections in the road network; *T* is the number of running periods of the charging network; *N*_*EV*_ is the total number of EVs contained in the road network; *n*_*m*_ and *N*_*m*_ are the nodes *n*_*m*_ and the total number of nodes contained in the service range with the *m*th charging station respectively, *n*_*m*_∈*N*_*m*_; *n*_*m*,*t*_ is the traffic flow of road network node *n* at time *t*.

The number of charging stations directly affects users’ demand for electric energy. When the road network contains charging stations of a certain scale, users’ electricity anxiety behavior will be greatly alleviated psychologically [34]. The influence of the number of charging stations on *S*_*EV*_ is as follows.

$$S_{sl}=\{\begin{array}{c} 0.7\;0.05\leq k_{m}<0.10\\ 0.5\;0.10\leq k_{m}<0.15\\ 0.3\;0.15\leq k_{m}<0.20 \end{array}$$ (15)

where *S*_*sl*_ is the effect of the number of charging stations on EV demand satisfaction; *k*_*m*_ is the scale of charging stations in the road-electric coupling network.

EV demand satisfaction is expressed as follows.

$$S_{EV}=\frac{1}{\vartheta S_{sl}+(1-\vartheta )S_{gld}}$$ (16)

where *ϑ* is the weight of quantitative factors in EV demand satisfaction.

#### 4.1.2 Average charging time

Average charging time *T*_*s*_ refers to the time when an EV enters a charging station until it finishes charging, which consists of queuing time and charging time.

$$T_{s}=\bar{T_{w}}+\frac{1}{N_{EV}}\sum_{i=1}^{N_{EV}}T_{c,i}$$ (17)

where *T*_*c*,*i*_ is the charging time of user *i*.

#### 4.1.3 EV charging cost

EV charging cost *C*_*EV*_ consists of electricity fee and charging service fee. The charging charge is determined jointly by the power grid price and charging quantity, and the charging service fee is determined by the charging network operator.

$$C_{EV}=\frac{1}{N_{EV}}\sum_{i=1}^{N_{EV}}(C_{e}+C_{s})\Delta E_{i}$$ (18)

where *C*_*e*_ is the electricity price of the power grid; *C*_*s*_ is the charging service fee; Δ*E*_*i*_ is the charging quantity of user *i*.

### 4.2. Indicators for evaluating the operation of charging networks

In terms of charging network operation, the main consideration is the spatial and temporal characteristics and economics of the stations, based on which evaluation indexes such as station utilization rate, service scope, and annual construction and operation cost are constructed.

#### 4.2.1. Charging station utilization rate

Charging station utilization rate *η*_*cs*_ refers to the ratio of the actual total time of charging service provided by each station to the total time of operation.

$$\eta_{cs}=\sum_{}\frac{t_{j}}{T_{j}}$$ (19)

where *t*_*j*_ is the total time that charging station *j* provides charging service to users; *T*_*j*_ is the business hours of charging station *j*.

#### 4.2.2. Scope of services

The scope of service *R* evaluates the service capability of the charging network from a spatial perspective, and indicates the service scope of the charging network by calculating the attractiveness of different charging stations to EVs.

$$\{\begin{array}{c} R=\sum_{m=1}^{M}\frac{\sqrt{\frac{1}{M}\sum_{m=1}^{M}{\left(R_{m}-\frac{1}{M}\sum_{m=1}^{M}R_{m}\right)}^{2}}}{R_{m}-\frac{1}{M}\sum_{m=1}^{M}R_{m}}\\ R_{m}=\frac{P_{m}}{k_{u}(\sum_{h=1}^{I}d_{h,l}\cdot \Delta Cap\cdot C_{cnep})} \end{array}$$ (20)

where *R*_*m*_ is the attractiveness of charging station m to users; the smaller *R*, the stronger the attractiveness of the charging station to EVs, and the larger the corresponding service range of the charging network; *P*_*m*_ is the operating power of the *m*th charging station; *k*_*u*_ is the influence weight of node *u*; *d*_*h*,*l*_ is the length of the road section of the EV to the charging station; *C*_*cnep*_ is the unit price of electricity sold by the charging network, where *C*_*cnep*_ = *C*_*e*_+*C*_*s*_.

#### 4.2.3. Annual construction and operating costs

This indicator reflects whether the planning of the charging network is economically rational. Annual construction and operation cost of charging network *C*_*CN*_ consists of annual construction cost and annual operation cost. Among them, the annual construction cost consists of the investment cost of the station’s distribution transformer, charger, land, and supporting equipment; the annual cost of operation and maintenance of the charging network is mainly composed of equipment depreciation, maintenance, and repair costs and labor costs. In general, the expenses are not very clear and can be solved as a percentage of the initial investment cost.

$$C_{CN}=(\frac{r_{0}{\left(1+r_{0}\right)}^{z}}{{\left(1+r_{0}\right)}^{z}‐1}+k_{ser})\sum_{i=1}^{M}\left(a_{T,i}N_{T,i}+b_{CD}N_{i}+s_{i}\right)$$ (21)

where *r*_0_ is the discount rate; *z* is the operating life of the charging network; *k*_*ser*_ is the conversion coefficient; *a*_*T*,*i*_ and *N*_*T*,*i*_ are the unit price of the transformer connected to charging station *i* and its quantity, respectively; *b*_*CD*_ is the unit price of the charging pile; *N*_*i*_ is the number of charging piles configured in the *i*th charging station; *s*_*i*_ is the infrastructure cost of charging station *i*.

### 4.3. Indicators for evaluating the impact of road network operation

The indicators of road network vulnerability, carbon emission reduction income and the amount of change in the road network operation index are constructed to assess the impact of charging network operation on the road network in terms of safety, economy, and congestion.

#### 4.3.1. Road network vulnerability

Road network vulnerability *V* refers to the average travel time variable of all vehicles in the road network when congestion occurs in the network. The higher the value of the indicator, the more congestion on the road section, which means the more fragile the overall road network is. This indicator can reflect the vulnerability status of the road network under the actual travel load. The formula for calculating the vulnerability of the road network is as follows.

$$V=\frac{\sum t_{rs}^{i}\cdot Q_{rs}}{\sum Q_{rs}}-\frac{\sum t_{rs}^{0}\cdot Q_{rs}}{\sum Q_{rs}}$$ (22)

where $t_{rs}^{i}$ is the shortest travel time of a vehicle from node *r* to *s* when road section *i* is congested; $t_{rs}^{i}$ is the shortest travel time of node *r* to *s* when the road section is unblocked; *Q*_*rs*_ is the traffic flow from node *r* to *s*.

#### 4.3.2. Carbon emission reduction income

EVs are powered by electricity and usually produce carbon emissions during the operation phase, but the source of carbon emissions is on the generation side. In the process of electric energy production, the carbon emission of electric energy mainly depends on the power generation structure and the share of power generation methods. Compared to the carbon emissions of conventional fuel vehicles, the carbon emissions of EVs are significantly reduced. Different charging networks have different acceptance capacity for EVs, different intensity of carbon emission reduction, and different corresponding carbon emission reduction benefits. The construction of carbon emission reduction benefit indicators can more intuitively present the contribution of charging network to environmental improvement.

$$\left\{ \begin{array}{l} M_{EV}=(M_{ele}\cdot E_{Tk})/\eta_{g\mathrm{rid}}\\ M_{ele}=(E_{pr}\sum_{i=1}^{5}\alpha_{i}\cdot m_{i})/E_{pr}\\ R_{T}=(M_{car}-M_{EV})\cdot \Delta l\cdot P_{T} \end{array}\right.$$ (23)

where *M*_*EV*_ is the carbon emission factor of EV; *M*_*ele*_ is a carbon emission factor for electrical energy; *η*_*grid*_ is the grid transmission efficiency; *E*_*pr*_ is the total regional electricity generation; *α*_*i*_ denotes the proportion of generation mode *i* to total generation; *m*_*i*_ denotes the carbon emission factor of the *i*th power generation mode, and the five power generation modes considered in this paper are coal power, natural gas power, hydro power, wind power and photovoltaic power; *P*_*T*_ is the carbon emission trading price; *R*_*T*_ is the carbon reduction revenue.

#### 4.3.3. Change amount of road network operation index

The charging demand of EVs and the operation condition of the charging network will cause sudden changes in the road condition of the traffic network, and the urban traffic performance index (TPI) is used to reflect the comprehensive operation condition of the road network. The correspondence between TPI and traffic speed is shown in Table 1. Change amount of road network operation index ΔTPI is the variation amount of operation index of all road sections in the network caused by the operation of the charging network.

$$\Delta TPI=\sum_{t=1}^{T}\sum_{i=1}^{I}\left(TPI_{i,t}-TPI_{i,t,0}\right)$$ (24)

where *TPI*_*i*,*t*,*0*_ and *TPI*_*i*,*t*_ are the road network operation indices before and after the charging network operation, respectively.

**Table 1:** Conversion relationship between TPI and traffic speed.

**TPI**	[0,2)	[2,4)	[4,6)	[6,8)	[8,10]
**Speed/(km∙h** ^**-1**^ **)**	>40	(30,40]	(20,30]	(15,20]	<15

### 4.4. Indicators for evaluating the impact of power grid operation

Unbalanced access to large-scale EVs will lead to node voltage reduction in the distribution network, which can lead to voltage collapse in severe cases [35]. According to the principles of economy, safety, and stability of distribution network, this paper constructs indicators such as network loss, voltage deviation non-out of limit rate, network security operation index, and variation of load peak-valley difference amount to evaluate the impact caused by the operation of the charging network on the distribution network.

#### 4.4.1. Network loss

Network Loss *E*_*loss*_ is a metric that considers the operation of the distribution network from an economic point of view. Due to the randomness of EV charging behavior, the operation of the charging network will change the power flow distribution of the power grid, and the network loss will also change accordingly.

$$E_{loss}=\sum_{}^{}G_{ij}(U_{i}^{2}+U_{j}^{2}-2U_{i}U_{j}\cos(\theta_{i}-\theta_{j}))$$ (25)

where *G*_*ij*_ is the conductance of line *ij*; *U*_*i*_ and *U*_*j*_ are the voltage amplitude values of the nodes *i* and *j*, respectively; *θ*_*i*_ and *θ*_*j*_ are the voltage phase angles of nodes *i* and *j*, respectively.

#### 4.4.2. Voltage deviation non-out of limit rate

The ratio of voltage nodes that do not cross the limit to the total number of nodes when the distribution network is connected to the load of the charging network. This indicator reflects the voltage fluctuations at the power grid nodes caused by the operation of the charging network.

$$\sigma =\frac{N_{v}}{N}\times 100\%$$ (26)

where *N*_*v*_ is the number of nodes in the distribution network that satisfy the voltage offset; *N* is the total number of nodes in the distribution network.

#### 4.4.3. Network security operation index

Network Security Operating Index *S* indicates the ratio of the number of lines with current overload to the total number of lines. This indicator is used to evaluate whether a certain line in the network meets the safe operation standard after the power grid is connected to the charging load.

$$S=\frac{L_{out}}{L}\times 100\%$$ (27)

where *L*_*out*_ is the number of lines in the distribution network that exceed the maximum safe operating current; *L* is the total number of lines in the distribution network.

#### 4.4.4. Variation of load peak-valley difference

Variation of load peak-to-valley difference Δ*P* is used to measure the impact of EV charging load on the grid load characteristics.

$$\Delta P=(P_{\max}-P_{\min})-(P_{\max,0}-P_{\min,0})$$ (28)

where *P*_*max*_ and *P*_*min*_ are the peak and valley values of the daily load curve of the power grid after charging network operation; *P*_*max*,*0*_ and *P*_*min*,*0*_ are the peak and valley values of the daily load curve of the power grid before the charging network operation, respectively.

## 5. Comprehensive evaluation of charging network based on TOPSIS

Based on the comprehensive evaluation system, AHP is used to determine the subjective weights of the criterion layer according to the subjective wishes of different decision-makers. The entropy weight method combined with the indicator values is used to determine the objective weights of the indicator layer. Finally, the improved TOPSIS evaluation model is used to comprehensively evaluate the criterion and objective layers and compare the advantages and disadvantages of the charging network planning schemes.

### 5.1. Determination of subjective weights at the criterion layer

In the comprehensive evaluation of the charging network, AHP is used to determine the weights of the four criterion layers, and the square root method is used in this paper to calculate the values of the criterion layer weights, which are calculated as follows.

$$w_{j}=\frac{\sqrt[{n}]{\prod_{y=1}^{n}a_{xy}}}{\sum_{x=1}^{n}\sqrt[{n}]{\prod_{y=1}^{n}a_{xy}}}$$ (29)

where *a*_*xy*_ is the comparison result of the importance of factor *x* and factor *y* in the AHP judgment matrix; *n* is the order of the judgment matrix.

The weight vector can be expressed as *Wa = (w*_*j*_*)*_*1*×*q*_, and the consistency test is performed on the judgment matrix to check whether it has logical rationality.

### 5.2. Determination of objective weights at the index layer

The entropy method is an objective empowerment method, which can be used to determine the degree of dispersion of an event by calculating the entropy value. When the same indicator has a large difference under different schemes, the indicator contains a larger amount of information and the corresponding weight is larger. Suppose that there are *m* evaluation programs, and the comprehensive evaluation system has *q* criterion layers and *n* evaluation indicators. The entropy weighting method is used to determine the indicator weight vector *W*_*b*_ under each criterion layer. The specific method is as follows.

Step 1: Construct the decision matrix. Construct a decision matrix *X*_*mn*_ based on the original index data under different schemes.

Step 2: Standardize indicators. Indicators are divided into positive indicators and negative indicators. Because different indicators have different scales and orders of magnitude, it is necessary to standardize the indicators. The processing methods of positive and negative indicators are as follows.

$$y_{ijk}=\frac{x_{ijk}-x_{\min,i}}{x_{\max,i}-x_{\min,i}}$$ (30)

$$y_{ijk}=\frac{x_{\max,i}-x_{ijk}}{x_{\max,i}-x_{\min,i}}$$ (31)

where *x*_*max*,*i*_ and *x*_*min*,*i*_ are the maximum and minimum values of indicator *i*, respectively; *x*_*ijk*_ is the actual value of indicator *i* in scheme *k*; *y*_*ijk*_ is the normalized value of indicator *i* in scheme *k*.

Step 3: Calculate the information entropy *e*_*ij*_ of each indicator.


$$p_{ijk}=\frac{y_{ijk}+1}{\sum_{k=1}^{m}\left(y_{ijk}+1\right)}$$ (32)

$$e_{ij}=-{\left(\ln\;m\right)}^{-1}\sum_{k=1}^{m}p_{ijk}\ln\;p_{ijk}$$ (33)

Step 4: Solve the weight *w*_*i*_ of indicator *i* and generate the weight vector *W*_*b*_ of the index layer.

$$\left\{ \begin{array}{l} w_{i}=\frac{1-e_{ij}}{\sum_{i=1}^{q_{j}}\left(1-e_{ij}\right)}\\ W_{b}={\left(w_{i}\right)}_{1\times q_{j}} \end{array}\right.$$ (34)

where *q*_*j*_ is the number of indicators under criterion layer *j*.

### 5.3. Comprehensive evaluation process

According to the criterion layer weight vector *W*_*a*_, index layer weight vector *W*_*b*_ and standardized data under different schemes, the improved TOPSIS evaluation model was used to determine the evaluation scores. The specific assessment process is as follows.

Step 1: Construct the weighted criteria matrix. The decision matrix *X* is normalized to obtain the matrix *R*, and the solved weights are multiplied with the matrix *R* to obtain the weighted criterion matrix *Z* [36].


$$Z={\left(z_{ij}\right)}_{m\times n}={\left(w_{j}r_{ij}\right)}_{m\times n}$$ (35)

Step 2: Determine the ideal solution. The positive and negative ideal solutions *z*^*+*^ and *z*^*-*^ are determined by the weighted standard matrix, respectively. The calculation method is shown below.


$$\left\{ \begin{array}{l} z_{j}^{+}=\underset{i}{\max}\;z_{ij}\\ z_{j}^{-}=\underset{i}{\min}\;z_{ij} \end{array}\right.$$ (36)

Step 3: Calculate the closeness of the charging network planning scheme to the positive and negative ideal solutions. The Euclidean distance and gray correlation are solved separately, and the two are combined to calculate the evaluation scores of each charging network solution with positive and negative ideal solutions.

Euclidean distance: for calculating the distance between different schemes and the ideal solution.


$$\{\begin{array}{c} D_{i}^{+}={\left(\sum_{j=1}^{n}{\left(z_{ij}-z_{j}^{+}\right)}^{2}\right)}^{\frac{1}{2}}\\ D_{i}^{-}={\left(\sum_{j=1}^{n}{\left(z_{ij}-z_{j}^{-}\right)}^{2}\right)}^{\frac{1}{2}} \end{array}$$ (37)

Gray correlation analysis method: to judge the degree of correlation of solutions by analyzing the development trend of different schemes. The gray correlation coefficient is solved as follows.

$$\{\begin{array}{c} v_{ij}^{+}=\frac{\underset{i}{\min}\underset{j}{\min}|z_{i}^{+}-z_{ij}|+\rho \underset{i}{\max}\underset{j}{\max}|z_{i}^{+}-z_{ij}|}{|z_{i}^{+}-z_{ij}|+\rho \underset{i}{\max}\underset{j}{\max}|z_{i}^{+}-z_{ij}"}\\ v_{ij}^{-}=\frac{\underset{i}{\min}\underset{j}{\min}|z_{i}^{-}-z_{ij}|+\rho \underset{i}{\max}\underset{j}{\max}|z_{i}^{-}-z_{ij}|}{\left|z_{i}^{-}-z_{ij}|+\rho \underset{i}{\max}\underset{j}{\max}|z_{i}^{-}-z_{ij}\right|} \end{array}$$ (38)

where *ρ* is the resolution factor and takes the value range [0,1].

Gray correlation:

$$\{\begin{array}{c} V_{i,j}^{+}=\frac{1}{m}\sum_{i=1}^{m}v_{ij}^{+}\\ V_{i,j}^{-}=\frac{1}{m}\sum_{i=1}^{m}v_{ij}^{-} \end{array}$$ (39)

Combining the Euclidean distance with the gray correlation to form a comprehensive assessment method. The positive and negative integrated ideal distances are shown in Eq (40).

$$\left\{ \begin{array}{l} S_{i}^{+}=k_{1}D_{i}^{-}+k_{2}V_{i,j}^{+}\\ S_{i}^{-}=k_{1}D_{i}^{+}+k_{2}V_{i,j}^{-} \end{array}\right.$$ (40)

where *k*_*1*_ and *k*_*2*_ are preference coefficients for the decision maker’s assessment.

In the positive integrated ideal distance $S_{i}^{+}$, when the negative Euclidean distance $D_{i}^{-}$ is larger and the positive gray correlation degree $V_{i,j}^{+}$ is higher, the closer the scheme to be evaluated is to the ideal solution, the higher the evaluation score will be. Conversely, the larger the negative integrated ideal distance $S_{i}^{-}$ is, the closer the evaluated scheme is to the negative ideal solution, which means that the charging network planning is worse under this scheme.

Step 4: Solve the integrated evaluation score. The positive and negative integrated ideal distances are combined to obtain the relative distances of different schemes from the integrated ideal solution. The calculation formula is as follows.


$$S_{i}=\frac{S_{i}^{+}}{S_{i}^{+}+S_{i}^{-}}$$ (41)

The higher the *S*_*i*_ value of the overall assessment score, the more reasonable the current charging network planning is. In the comprehensive evaluation of the target layer, according to the evaluation results of the criterion layer *S*_*i*,*c*_, and combined with the different weights of the four criteria by different decision-makers, the comprehensive evaluation score of the target layer *S*_*i*,*t*_ can be solved.

**Fig 4:**
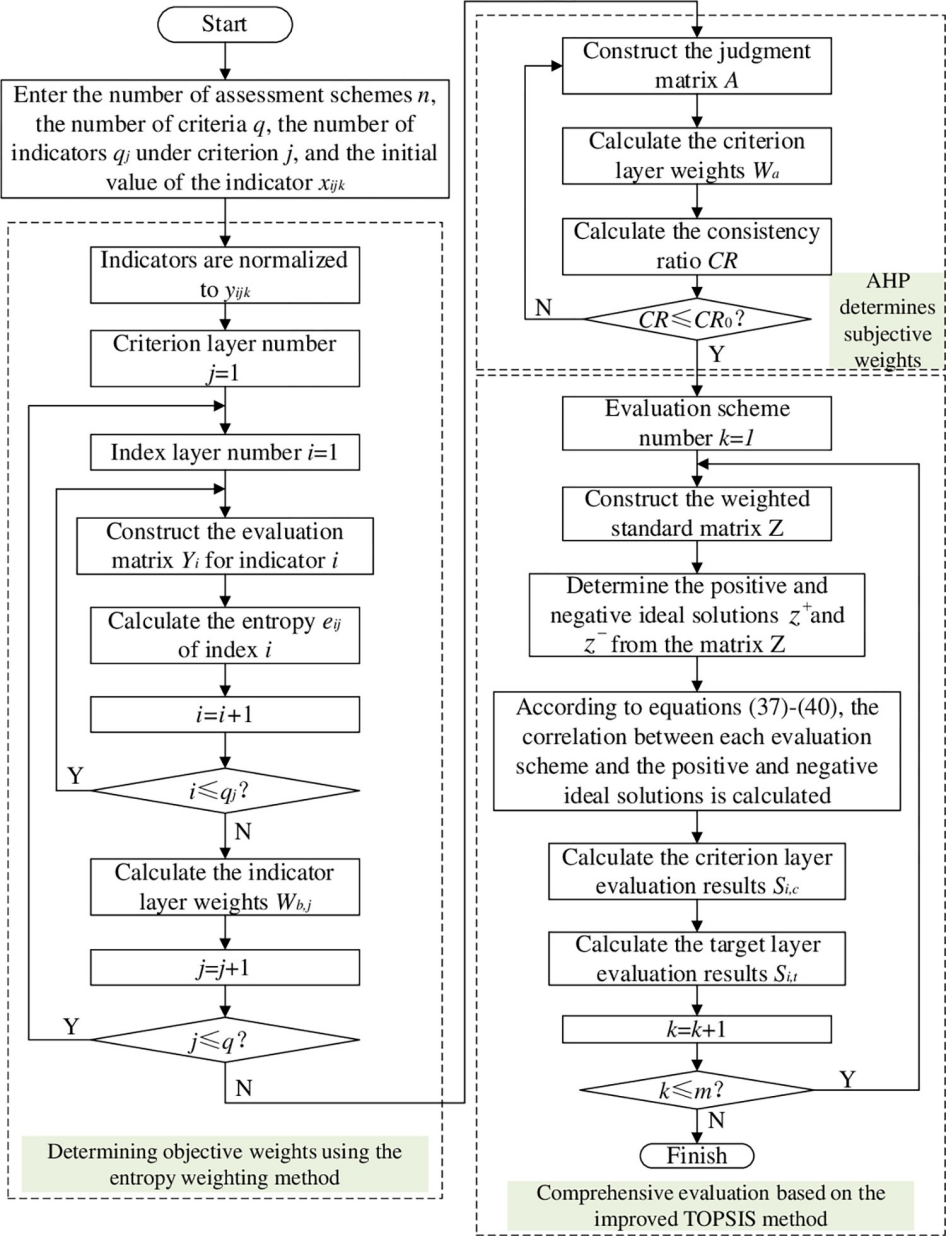
Charging network comprehensive assessment flow chart.

The comprehensive evaluation process of the EV charging network based on the improved TOPSIS evaluation method is shown in Fig 4.

## 6. Case study

### 6.1. Parameter settings

**Fig 5:**
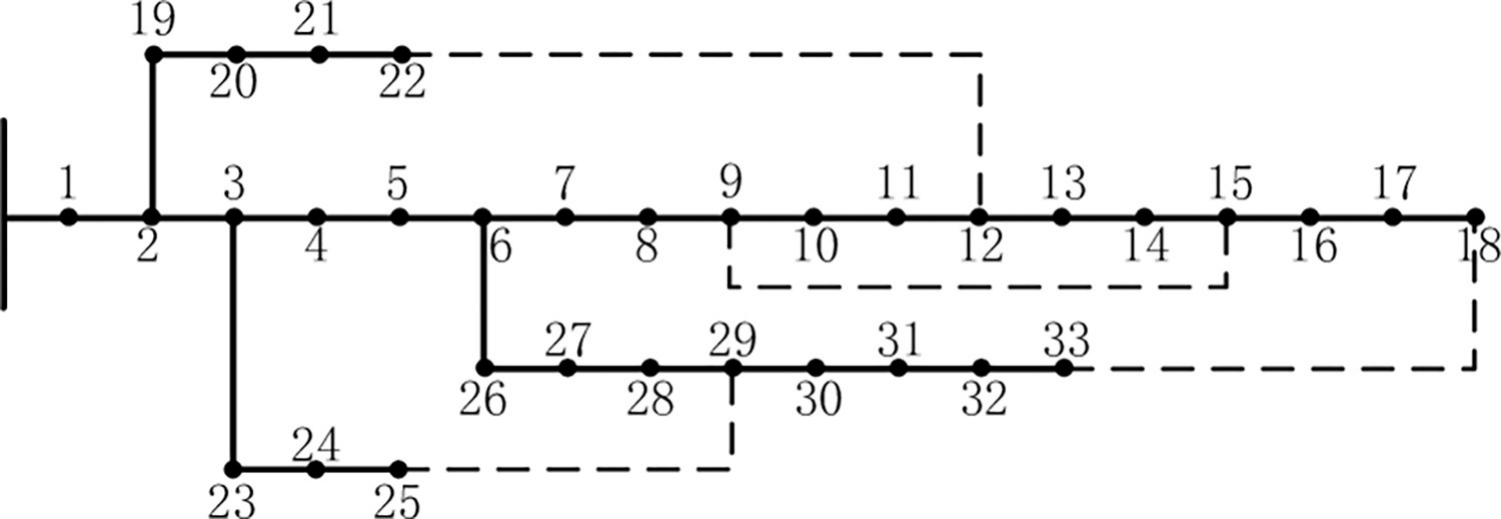
IEEE 33 nodes distribution network topology diagram.

This paper takes IEEE 33 nodes distribution network and regional traffic road network as an example for simulation analysis. Set the base power of distribution network as 10MV∙A, the base voltage as 12.66kV, and the topology of distribution network as shown in Fig 5.

**Fig 6:**
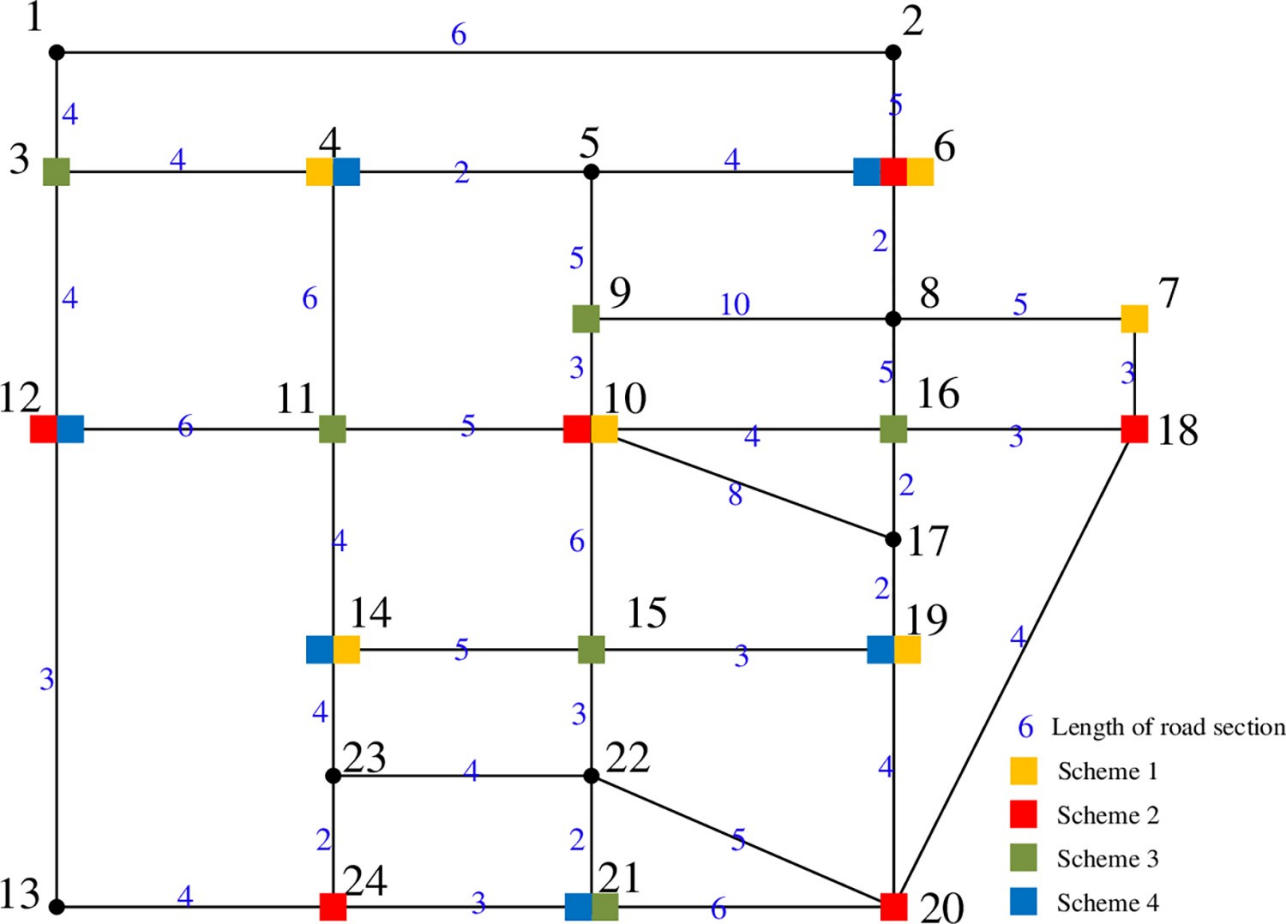
Regional road network topology and scheme layout.

Sioux Falls urban area traffic road network model is used in this paper, the network contains 24 road network nodes and 38 road sections, and the relevant parameters and saturation of the road sections are shown in Table A1 in S1 Appendix. It is assumed that the regional traffic road network contains 7,500 EVs, taking Tesla Model 3 as an example, and all road sections are set as Class I roads. Six charging stations are planned to be built in the current regional transportation network, and the EV charging mode is set to be fast charging, and the specific parameter settings are shown in Table A2 in S1 Appendix. This paper will make a comprehensive evaluation of four urban charging network planning schemes, and the distribution of charging station planning schemes and the topology of regional traffic road network are shown in Fig 6.

In the charging network planning scheme, charging stations are all set on the nodes of the road network. Charging stations of scheme 1 are distributed on nodes 4, 6, 7, 10, 14, and 19. In scheme 2, charging stations are distributed on nodes 6, 10, 12, 18, 20, and 24. Charging stations in scheme 3 are distributed on nodes 3, 9, 11, 15, 16, and 21. Charging stations in scheme 4 are distributed on nodes 4, 6, 12, 14, 19, and 21. The configuration of the number of charging piles for the four planning scenarios is shown in Table 2. The nodal correspondence between the traffic road network and the distribution network is shown in Table A3 in S1 Appendix.

**Table 2:** Configuration of charging piles.

Scheme	Charging station #1*	Charging station #2	Charging station #3	Charging station #4	Charging station #5	Charging station #6
Scheme 1	14	16	15	13	14	18
Scheme 2	13	18	15	17	11	16
Scheme 3	10	17	15	16	18	14
Scheme 4	15	16	12	17	16	14

* The serial numbers of charging stations of different schemes are sorted according to the road network nodes from smallest to largest.

### 6.2. Analysis of results

#### 6.2.1. Comprehensive evaluation at the criterion layer

According to the comprehensive evaluation system of charging network constructed in Section 3 of this paper, the data sources are obtained and the initial values of indicators for the four charging network planning schemes are calculated through simulation, and the initial values of indicators are standardized to form a standardized decision matrix, as shown in Tables A4 and A5 in S1 Appendix.

**Fig 7:**
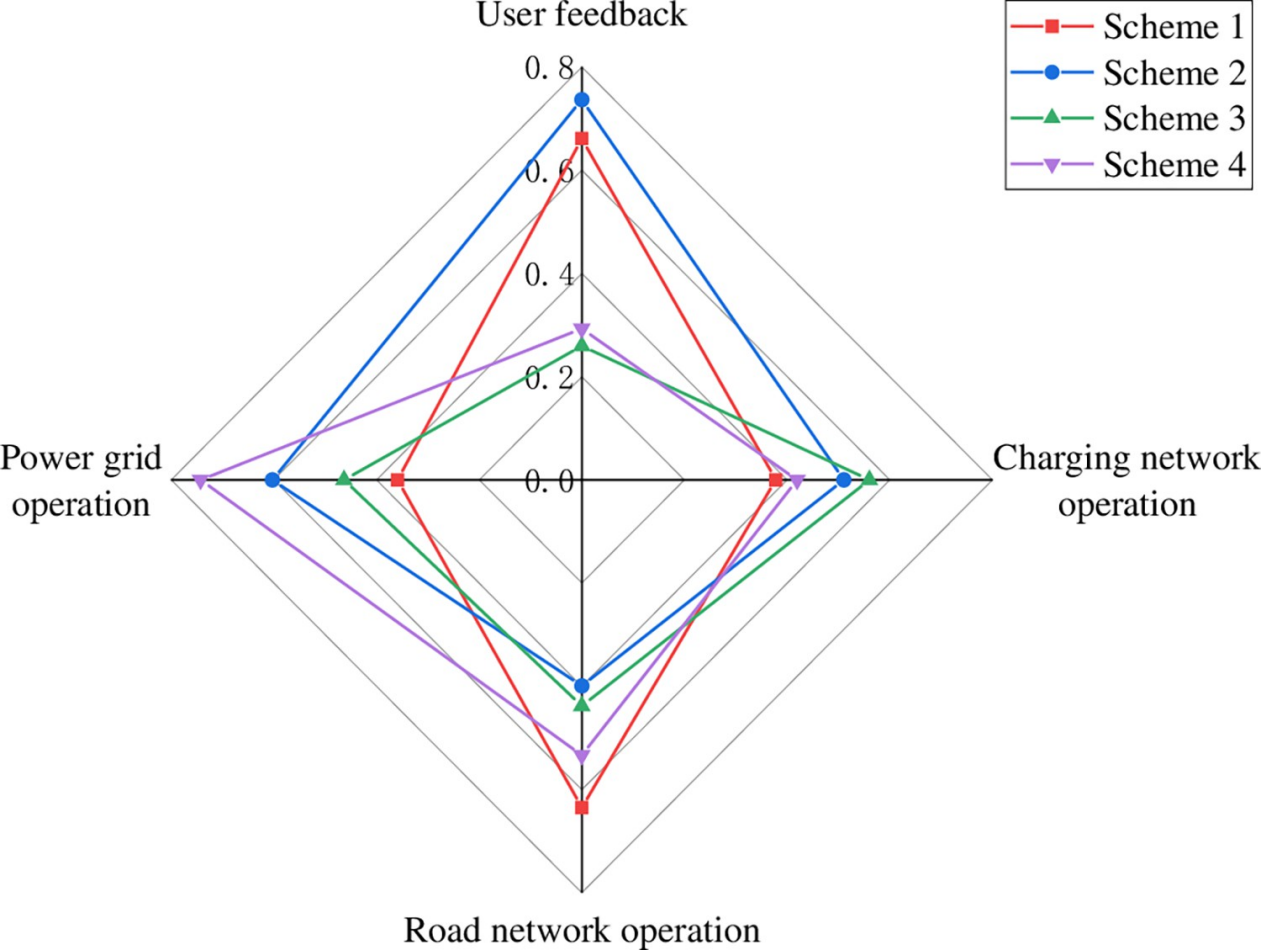
Radar chart of assessment results at the criterion layer.

The weights of each indicator layer were determined using the entropy weighting method as shown in Eq (42). Combined with the improved TOPSIS evaluation model for the comprehensive evaluation of the four charging network planning schemes, the results are shown in Fig 7. The evaluation results reflect the advantages and disadvantages of different planning schemes in four dimensions: user feedback, charging network operation, road network operation, and grid operation.


$$\left\{ \begin{array}{l} W_{b-c1}=(0.3198,0.3062,0.3740)\\ W_{b-c2}=(0.2883,0.3838,0.3279)\\ W_{b-c3}=(0.2918,0.3886,0.3196)\\ W_{b-c4}=(0.2315,0.2340,0.2797,0.2548) \end{array}\right.$$ (42)

From the evaluation results in Fig 7, it can be seen that scheme 1 is the best in terms of road network operation, but has the lowest rating in terms of grid operation and charging network operation, which is caused by unreasonable planning of charging stations, the low utilization rate of charging piles in some charging stations and small service area of charging stations. Scheme 2 performs best in terms of user feedback, but has the lowest rating in terms of road network operation. Scheme 3 has the best performance in charging network operation, but the lowest rating in user feedback, which is due to the unreasonable distribution location of charging stations, resulting in higher charging time and cost for users and lower user satisfaction. Scheme 4 showed the best performance in grid operation, but scored poorly in user feedback and charging network operation.

**Fig 8:**
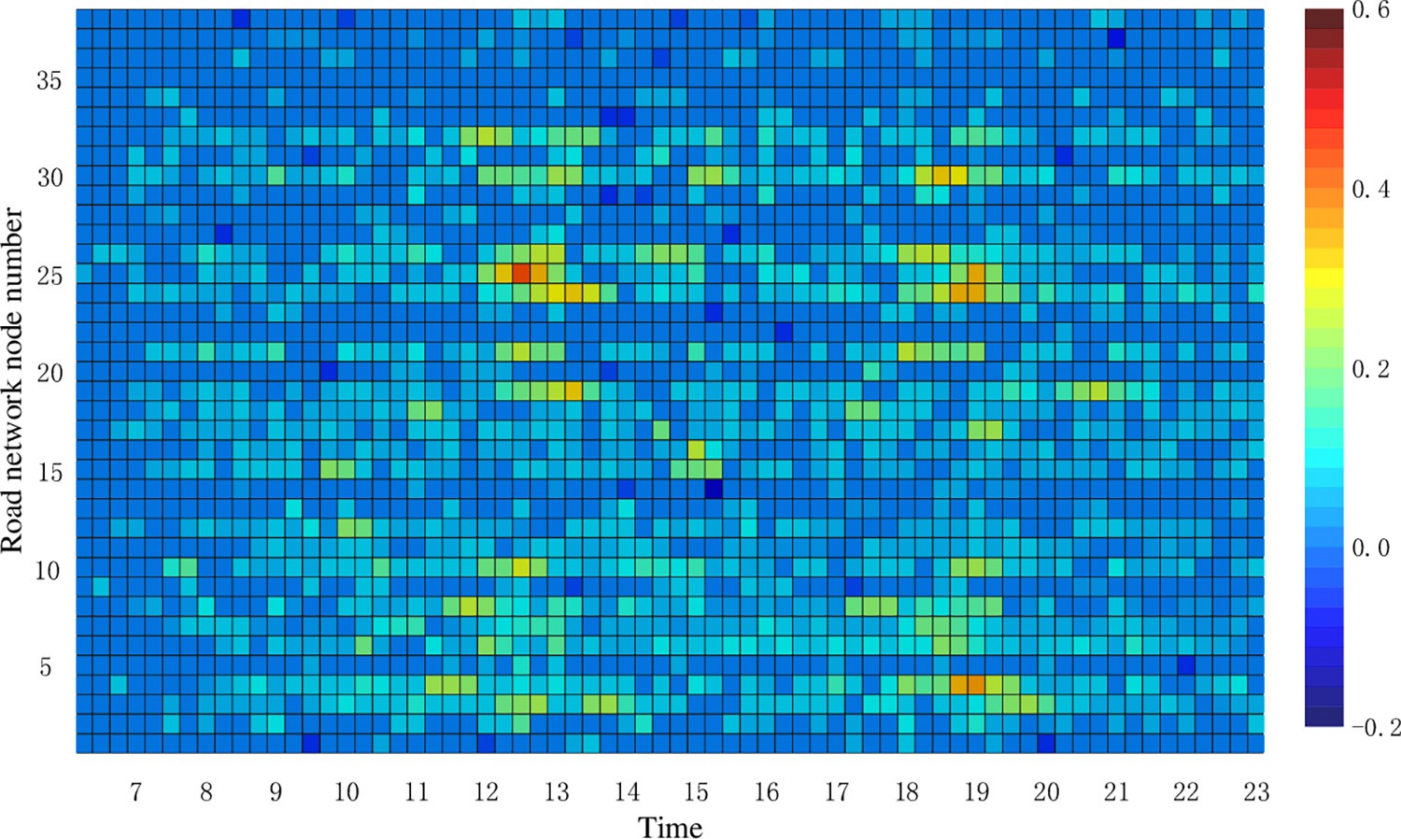
Thermogram of TPI changes for scheme 1.

**Fig 9:**
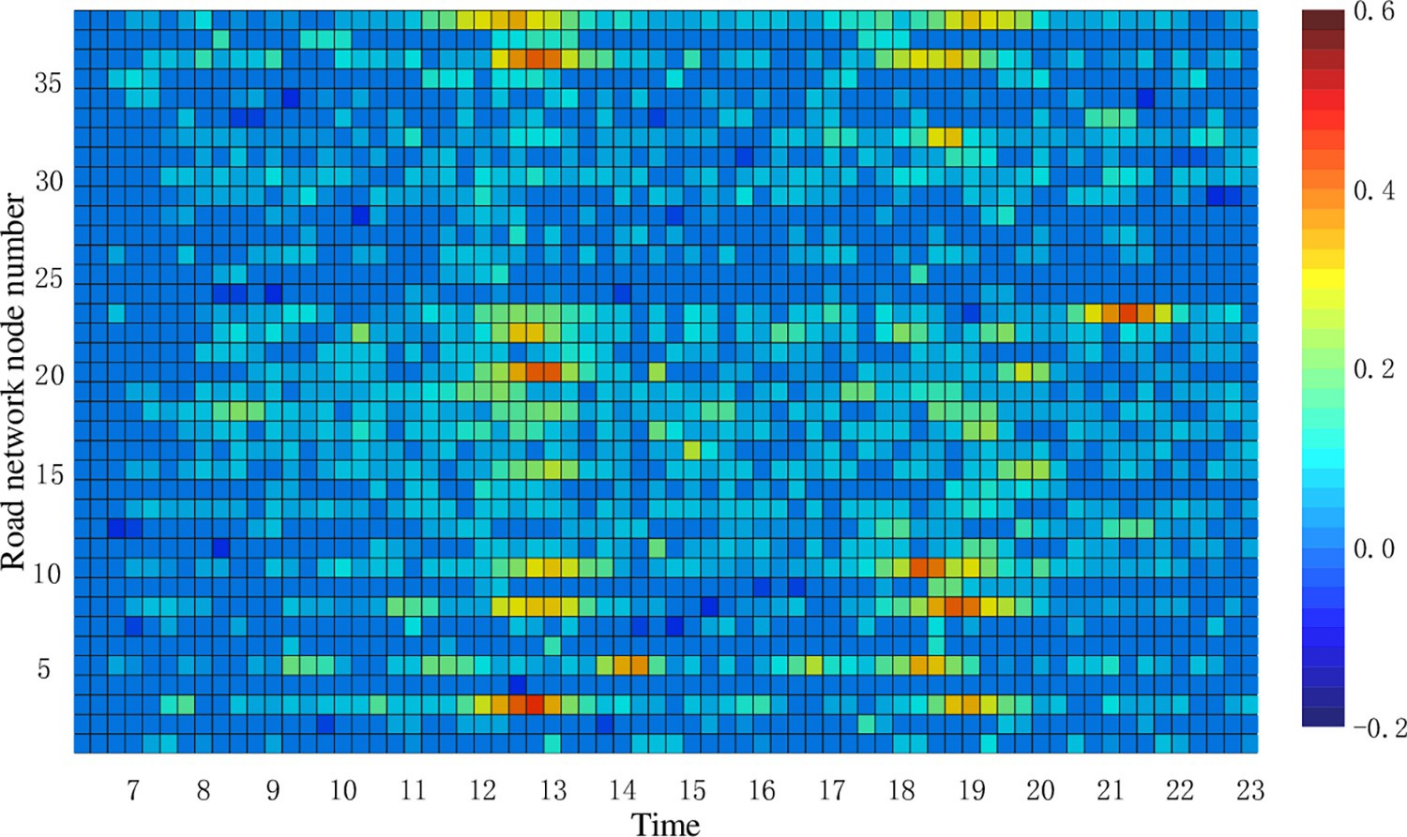
Thermogram of TPI changes for scheme 2.

In terms of road network operation impact, scheme 1 is significantly better than scheme 2. Scheme 1 has 4.17% less road network vulnerability than scheme 2, 6.6% more carbon emission reduction benefits than scheme 2, and 3.14 less road network operation index changes than scheme 2. This is caused by the different locations of charging stations in the road network for scheme 1 and scheme 2. Figs 8 and 9 show the heat map of TPI variation under the two charging network planning schemes, respectively.

From Figs 8 and 9, it can be seen that the overall TPI variation of scheme 1 is small compared to scheme 2. In scheme 1, the charging station arranged at node 14 has a greater impact on the TPI of sections 21, 24, and 25. In scheme 2, the charging station arranged at node 24 has a greater impact on the TPI of road sections 23, 36, and 38, while the charging station arranged at node 6 has the greatest impact on the TPI of road sections 3, 8, and 10.

#### 6.2.2. Comprehensive evaluation of target layer

AHP is used to construct a judgment matrix from the perspectives of decision-makers such as charging network operators, power supply companies, and transportation sector, and calculate the criterion layer weights of different decision-makers. The weight calculation results are shown in Table 3.

**Table 3:** Criteria layer indicator weights under different decision-makers.

Decision maker	User feedback	Charging network operation	Road network operation	Power grid operation
Charging network operators	0.290	0.542	0.103	0.065
Power supply company	0.136	0.214	0.064	0.586
Transportation sector	0.266	0.108	0.519	0.107

The improved TOPSIS evaluation model was used to calculate the weighted Euclidean distance, gray correlation, and comprehensive evaluation scores between different indicators and positive and negative ideal solutions under each scheme by combining the criteria layer weights of different decision-makers. The comprehensive evaluation results of the target layer under different decision-makers are shown in Table 4.

**Table 4:** Comprehensive evaluation results of the target layer under different decision-makers.

Decision maker	Scheme 1	Scheme 2	Scheme 3	Scheme 4
Charging network operators	0.509	0.576	0.454	0.453
Power supply company	0.438	0.556	0.430	0.559
Transportation sector	0.549	0.529	0.414	0.480

When charging network operation has the highest weight in the criterion layer, scheme 3 is the best in terms of charging network operation in the criterion layer evaluation, but has the lowest evaluation score in the target layer evaluation. The main reason is that scheme 3 is at a disadvantage in terms of user feedback, road network operation, and grid operation. At this time, the charging network planning preference is ranked as follows: Scheme 2 > Scheme 1 > Scheme 3 > Scheme 4.

When power grid operation has the highest weight in the criterion layer, scheme 4 has the most advantage in power grid operation in the criterion evaluation, and also has the highest overall score in the comprehensive evaluation of the target layer. When the power supply company is the main decision maker, the charging network planning scheme is ranked: Scheme 4 > Scheme 2 > Scheme 1 > Scheme 3.

When the road network operation has the highest weight in the criterion layer, scheme 1 is significantly better than the other three schemes in the comprehensive evaluation of the criterion layer in terms of road network operation, and also has the highest comprehensive score in the target layer. When the transportation department is the main decision maker, the order of planning options: Scheme 1 > Scheme 2 > Scheme 4 > Scheme 3.

The evaluation results of planning scheme will be different when different decision-making subjects conduct comprehensive evaluation of target layer. Because different decision-makers have subjective willingness in considering the weight of the criterion layer, which leads to different evaluation results.

#### 6.2.3. Improvement of charging network planning scheme

**Fig 10:**
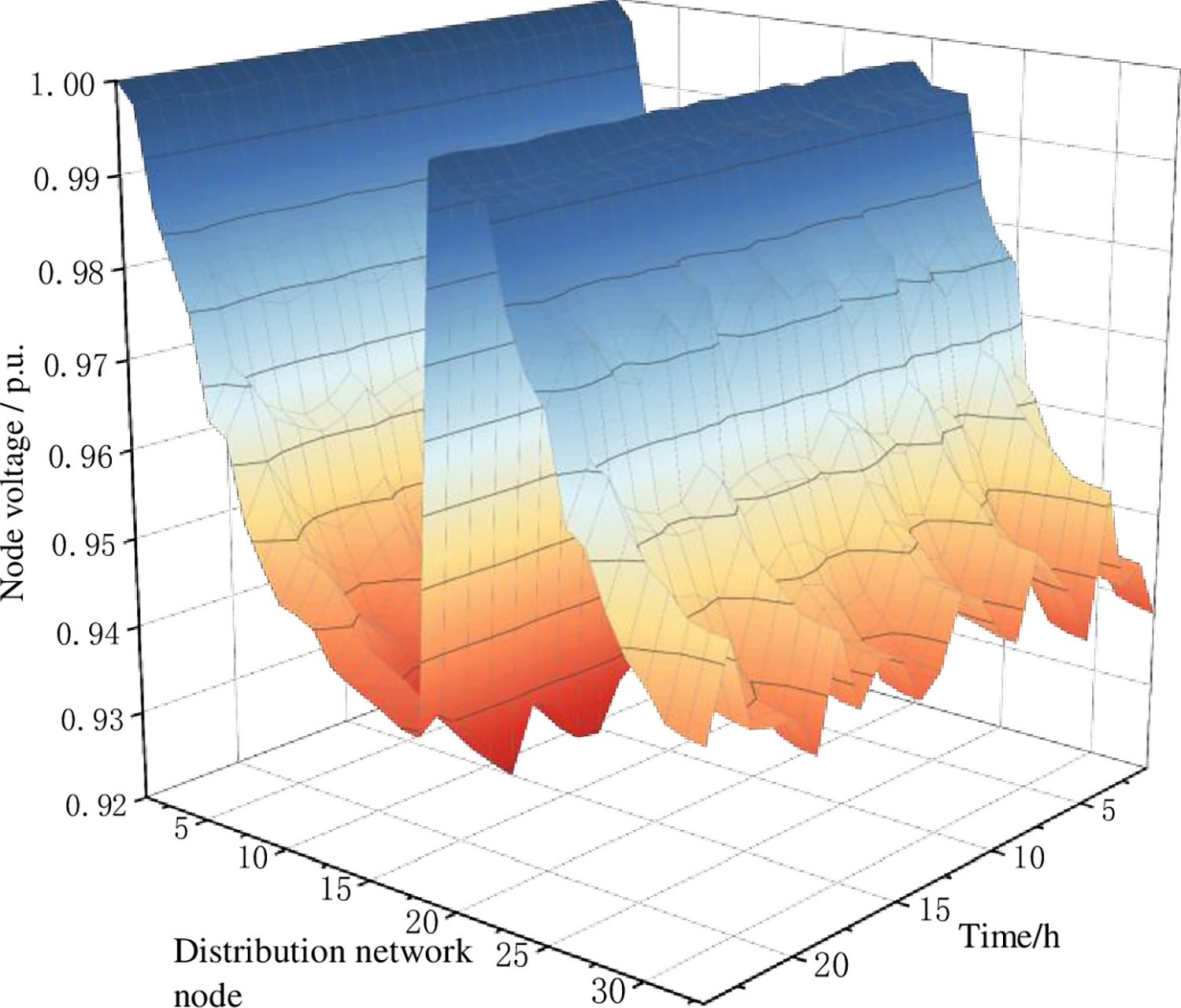
The node voltage level without access to the charging network.

**Fig 11:**
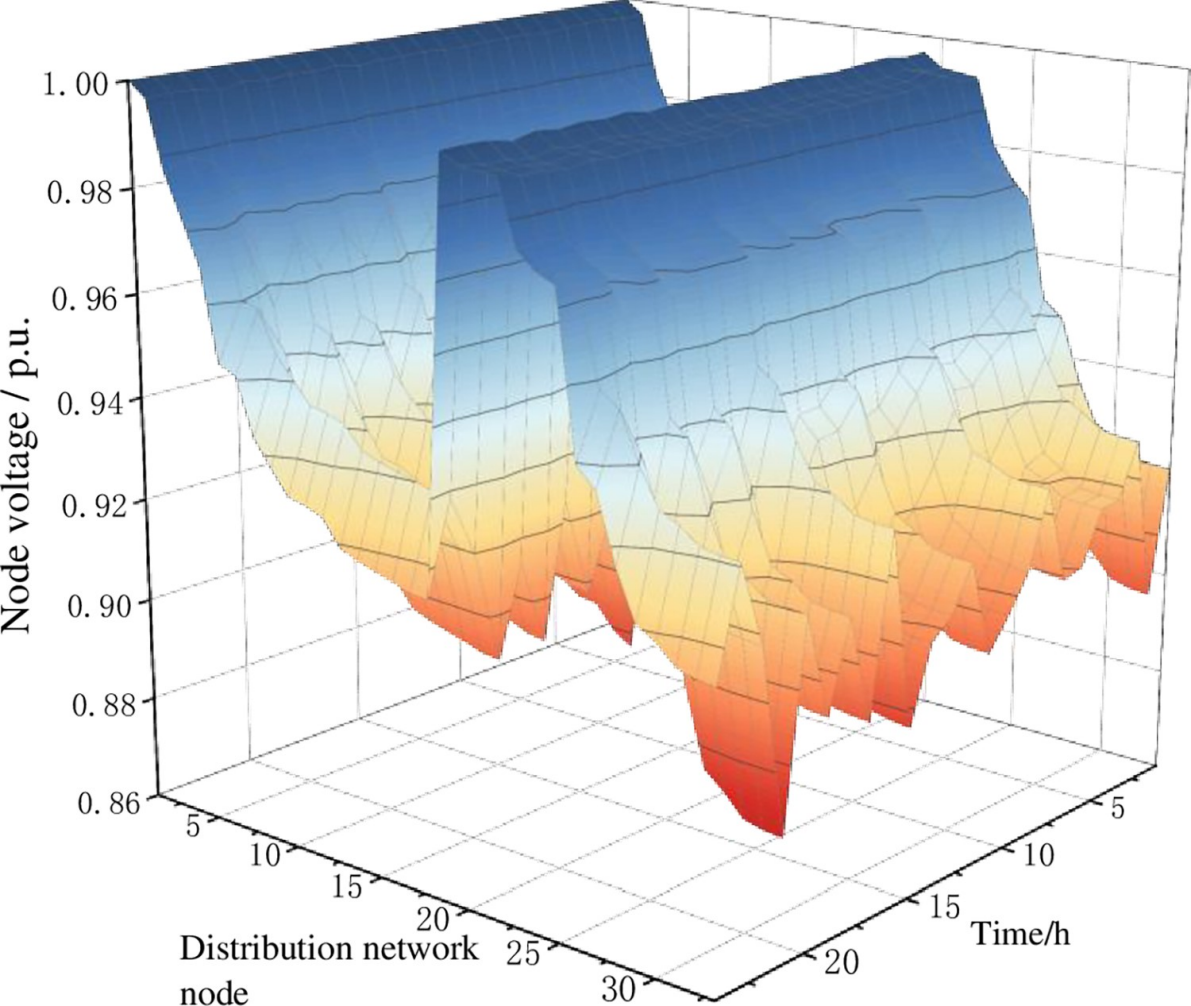
The node voltage level under charging network scheme 1.

In the criterion layer evaluation, scheme 1 has the lowest score result in terms of power grid operation impact and charging network operation. It is mainly at the power grid operation criterion layer that scheme 1 performs worse in terms of voltage deviation non-out of limit rate and network security operation index, resulting in a larger gap in evaluation scores compared to other schemes. At the charging network operation level, there is a situation of low station utilization. Figs 10 and 11 show the node voltage distribution before and after the charging network planning scheme 1 is connected to the distribution network operation, respectively.

When the charging network of planning scheme 1 is connected, the distribution network node 29 (access to the charging station at road network node 7) is located at the back of the network and the minimum voltage drops from 0.925 p.u. to 0.897 p.u. during the period of 13:00–15:00. During the 14:00–15:00 time period, nodes 29–33 of the distribution network experience a serious limit crossing, which threatens the safe operation of the distribution network. During the period 18:00–19:00, the voltage at distribution network node 13 (access to the charging station at road network node 19) dropped from 0.930 p.u. to 0.907 p.u. and no overrun occurred at the subsequent node of the branch road where it was located.

From Fig 8, it can be seen that road sections 26, 30, and 32 around road network node 19 have different degrees of congestion during the 18:00–19:00 hours. In the subsequent charging station planning, the construction of charging stations on non-congested road sections can be considered, while charging network operators can adopt certain charging guidance strategies to alleviate the traffic pressure on congested road sections to enhance the feasibility of the charging network planning scheme.

**Fig 12:**
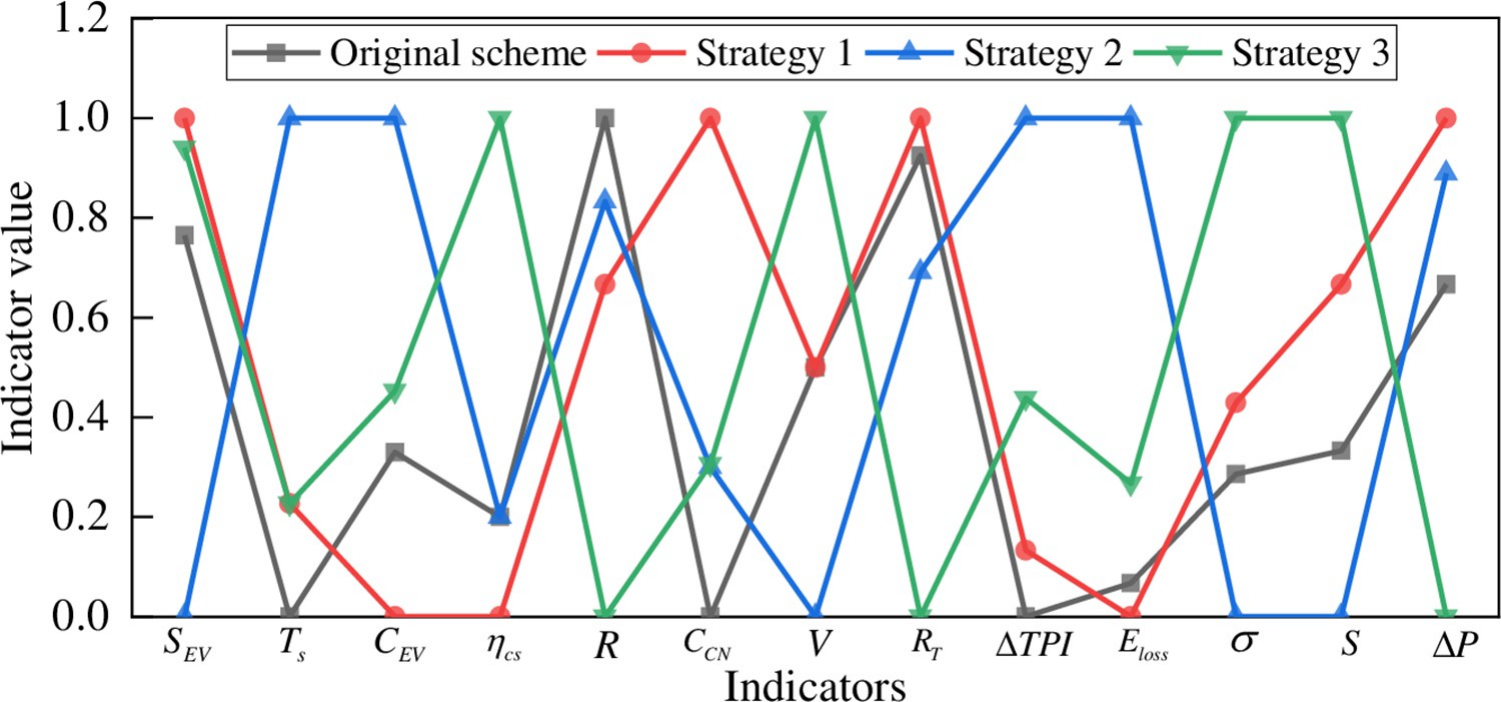
Standardized data for indicators.

**Fig 13:**
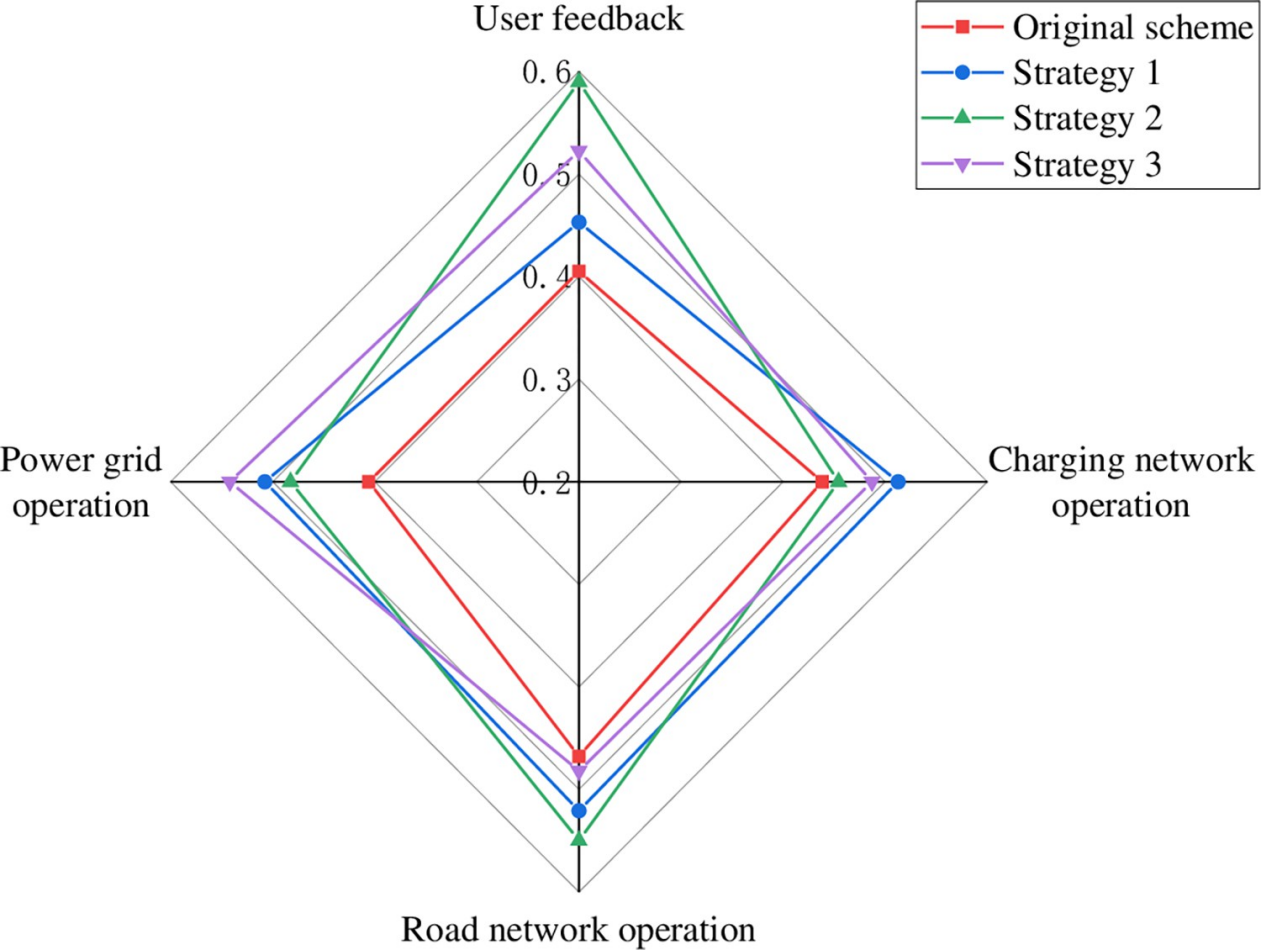
Comparison of results before and after adopting charging guidance strategy.

This paper adopts three charging guidance strategies to optimize and compare the charging network. Strategy 1: Propose a charging guidance strategy considering the scope of virtual services, and unify EV charging load in time transfer and space transfer to the same time scale to control [37]. Strategy 2: Minimize charging cost considering charging cost and time cost as the guiding goal [38]. Strategy 3: Real-time pricing strategy for charging stations considering virtual load and utilization balancing [39]. Scheme 1 is considered to be optimized using different charging strategies, and the standardized data of each index are shown in Fig 12. The results of solving the Euclidean distance and gray correlation are shown in Table A6 in S1 Appendix, and the results of the criterion level evaluation are shown in Fig 13.

As can be seen from Fig 13, considering the impact of different charging guidance strategies on the charging network planning scheme 1, different optimization strategies have different degrees of impact on each criterion layer of the planning scheme. In terms of power grid operation, the real-time pricing strategy used in strategy 3 is the most effective in optimizing power grid operation. In terms of user feedback, the use of strategy 2 improves more significantly compared to other strategies, with an improvement of 0.18 compared to the evaluation result of the original solution. This is because strategy 2 is guided by the goal of minimizing the charging cost and time cost for users, which significantly reduces the queuing time and charging cost for users, while user satisfaction is improved. The three guiding strategies have different degrees of improvement in the operation of charging network and road network.

## 7. Conclusions

In this paper, a comprehensive evaluation model of the EV charging network planning scheme under transportation network-grid coupling is proposed. A comprehensive evaluation system of the charging network is constructed from four aspects: user feedback, charging network operation, road network operation impact, and grid operation impact, and the improved TOPSIS method is used to make a comprehensive evaluation of the planning scheme. The conclusions obtained are shown as follows.

1. The comprehensive evaluation system proposed in this paper can analyze different charging network planning schemes. Analyzing the advantages and disadvantages of 4 aspects: users, charging network, road network, and power grid, provides reference opinions for the decision-making department. The carbon emission reduction benefit index is constructed to visually present the environmental feasibility of the planning scheme.
2. In the comprehensive evaluation process, this paper considers the subjective intentions of different decision-makers and the objective effects of indicators. It is also combined with the improved TOPSIS evaluation method to determine the evaluation results of the planning scheme.
3. The comprehensive evaluation system of the charging network can analyze the weaknesses in the daily operation process and provide the improvement basis for the subsequent continuous and stable operation of the charging network. At the same time, it promotes the coordinated and unified development of the road-electric coupling interaction network.

The comprehensive indicator system proposed in this paper involves charging network, users, transportation network and distribution network, with a large number of indicators and intertwined effects. Therefore, studying the correlation among indicators and screening the key indicators for charging network assessment will be the focus of subsequent research.

## Supporting information

S1 Appendix (DOCX)

## References

[1] Casals L.C.; Martinez-Laserna E.; Garcia B.A.; Nieto N. Sustainability analysis of the electric vehicle use in Europe for CO2 emissions reduction. *J*. *Clean Prod*. 2016, 127, 425–437. doi: 10.1016/j.jclepro.2016.03.120

[2] He X.Y.; Wu Y.; Zhang S.J.; Tamor M.A.; Wallington T.J.; Shen W.; et al. Individual trip chain distributions for passenger cars: Implications for market acceptance of battery electric vehicles and energy consumption by plug-in hybrid electric vehicles. *Appl*. *Energy* 2016, 180, 650–660. doi: 10.1016/j.apenergy.2016.08.021

[3] Madina C.; Zamora I.; Zabala E. Methodology for assessing electric vehicle charging infrastructure business models. *Energy Policy* 2016, 89, 284–293. 10.1016/j.enpol.2015.12.007

[4] Serradilla J.; Wardle J.; Blythe P.; Gibbon J. An evidence-based approach for investment in rapid-charging infrastructure. *Energy Policy* 2017, 106, 514–524. 10.1016/j.enpol.2017.04.007

[5] Shao S.N.; Pipattanasomporn M.; Rahman S. Challenges of PHEV Penetration to the Residential Distribution Network. In Proceedings of the General Meeting of the IEEE-Power-and-Energy-Society, Calgary, CANADA, Jul 26–30, 2009; pp. 2522–2529.

[6] Yang X.; Lu L.; Xiang Y.; Liu Y.; Liu F. Degradation charging scenarios and impacts on voltage stability of urban distribution network under "EV-road-grid"coupling. *Electr*. *Power Autom*. *Equip*. 2019, 39, 102–108 and 122.

[7] Chen J.; Huang X.Q.; Tian S.M.; Cao Y.J.; Huang B.C.; Luo X.Y.; et al. Electric vehicle charging schedule considering user’s charging selection from economics. *IET Gener*. *Transm*. *Distrib*. 2019, 13, 3388–3396. 10.1049/iet-gtd.2019.0154

[8] Chen L.; Zhang Y.; Figueiredo A. Charging load forecasting of electric vehicles based on multi-source information fusion and its influence on distribution network. *Electr*. *Power Autom*. *Equip*. 2018, 38, 1–10.

[9] Lin M.; Hu Z.; Gao M.; Chen J. Reliability Evaluation of Distribution Network Considering Demand Response and Road-Electricity Coupling Characteristics of Electric Vehicle Load. *Electr*. *Power Constr*. 2021, 42, 86–95.

[10] Wang Z.P.; Song C.B.; Zhang L.; Zhao Y.; Liu P.; Dorrell D.G. A Data-Driven Method for Battery Charging Capacity Abnormality Diagnosis in Electric Vehicle Applications. *IEEE Trans*. *Transp*. *Electrif*. 2022, 8, 990–999. 10.1109/tte.2021.3117841

[11] Zeng M.; Pan Y.F.; Zhang D.Y.; Lu Z.Y.; Li Y. Data-Driven Location Selection for Battery Swapping Stations. *IEEE Access* 2019, 7, 133760–133771. 10.1109/access.2019.2941901

[12] Wang S.Q.; Shao C.F.; Zhuge C.X.; Sun M.D.; Wang P.X.; Yang X. Deploying Battery Swap Stations for Electric Freight Vehicles Based on Trajectory Data Analysis. *IEEE Trans*. *Transp*. *Electrif*. 2022, 8, 3782–3800. 10.1109/tte.2022.3160445

[13] Gao Y.; Yang J.J.; Yang M.; Li Z.S. Deep Reinforcement Learning Based Optimal Schedule for a Battery Swapping Station Considering Uncertainties. *Ieee Transactions on Industry Applications* 2020, 56, 5775–5784. 10.1109/tia.2020.2986412

[14] Infante W.; Ma J.; Han X.Q.; Liebman A. Optimal Recourse Strategy for Battery Swapping Stations Considering Electric Vehicle Uncertainty. *Ieee Transactions on Intelligent Transportation Systems* 2020, 21, 1369–1379, 10.1109/tits.2019.2905898

[15] Mahoor M.; Hosseini Z.S.; Khodaei A. Least-cost operation of a battery swapping station with random customer requests. *Energy* 2019, 172, 913–921. 10.1016/j.energy.2019.02.018

[16] Yan X.; Zhao S.; Dong Q.; Wang L.; Liu Z.; Bai S.; et al. Comprehensive evaluation of electric vehicle charger performance. *Power Syst*. *Prot*. *Control* 2020, 48, 164–171.

[17] Sheng R.; Tang Z.; Xue J. Dynamic Evaluation Method of EV Charging Station Service Capability Under Multi Indicators EV. *Proc*. *CSEE* 2021, 41, 4891–4904.

[18] Andrenacci N.; Ragona R.; Genovese A. Evaluation of the Instantaneous Power Demand of an Electric Charging Station in an Urban Scenario. *Energies* 2020, 13. 10.3390/en13112715

[19] Liu W.; Wang Z.; Xiao T.; Lu C. AMI data-driven state evaluation method for measurement and operation error of electric vehicle charging facilities. *Electr*. *Power Autom*. *Equip*. 2022, *in press*.

[20] Zenginis I.; Vardakas J.; Zorha N.; Verikoukis C. Performance Evaluation of a Multi-Standard Past Charging Station for Electric Vehicles. *IEEE Trans*. *Smart Grid* 2018, 9, 4480–4489. 10.1109/tsg.2017.2660584

[21] Ucer E.; Koyuncu I.; Kisacikoglu M.C.; Yavuz M.; Meintz A.; Rames C. Modeling and Analysis of a Fast Charging Station and Evaluation of Service Quality for Electric Vehicles. *IEEE Trans*. *Transp*. *Electrif*. 2019, 5, 215–225. 10.1109/tte.2019.2897088

[22] Liu J.; Xiang Y.; Yao H.; Tang S.; Jawad S. Discussion on planning and operation of charging service network integrated with power and transportation networks. *Power Syst*. *Prot*. *Control* 2019, 47, 1–12.

[23] Nan Q.; Mu Y.; Dong X.; Jia H.; Zhou Y.; Xu Z. Comprehensive Evaluation Index System and Method for Fast Charging Network of Electric Vehicles. *Autom*. *Electr*. *Power Syst*. 2020, 44, 83–91.

[24] Wang M.; Xiang Y.; Zhou C.; Zhao H.; Liu J. Multi-dimension Evaluation Index System and Method of Urban Charging Service Network. *J*. *Global Energy Interc*. 2022, 5, 261–270.

[25] Zhou Z.C.; Dong X.J.; Wang C.L.; Wu G.; Xue Y.N.; Shi R.F. An Comprehensive Assessment Model for the Distribution Network with the New Type of Loads Based on the TOPSIS Method. In Proceedings of the 2nd IEEE Conference on Energy Internet and Energy System Integration (EI2), Beijing, PEOPLES R CHINA, Oct 20–22, 2018; pp. 651–655.

[26] Tang W.; Wu B.X.; Zhang L.; Zhang X.H.; Li J.X.; Wang L. Multi-objective Optimal Dispatch for Integrated Energy Systems Based on a Device Value Tag. *CSEE J*. *Power Energy Syst*. 2021, 7, 632–643. 10.17775/cseejpes.2019.02650

[27] Mei J.; Gao C. Considerations of traffic characteristics in research of grid integration of electric vehicles. *Power Syst*. *Technol*. 2015, 39, 3549–3555.

[28] Shao Y.; Mu Y.; Yu X.; Dong X.; Jia H.; Wu J.; et al. A Spatial-temporal Charging Load Forecast and Impact Analysis Method for Distribution Network Using EVs-Traffic-Distribution Model. *Proc*. *CSEE* 2017, 37, 5207–5219.

[29] Mu Y.F.; Wu J.Z.; Jenkins N.; Jia H.J.; Wang C.S. A Spatial-Temporal model for grid impact analysis of plug-in electric vehicles. *Appl*. *Energy* 2014, 114, 456–465. 10.1016/j.apenergy.2013.10.006

[30] Zhang M.; Sun Q.; Yang X. Electric Vehicle Charging Load Prediction Considering Multi-source Information Real-time Interaction and User Regret Psychology. *Power Syst*. *Technol*. 2022, 46, 632–641. 10.13335/j.1000-3673.pst.2021.0273

[31] Xing Q.; Chen Z.; Zhang Z.Q.; Xu X.; Zhang T.; Huang X.L.; et al. Urban Electric Vehicle Fast-Charging Demand Forecasting Model Based on Data-Driven Approach and Human Decision-Making Behavior. *Energies* 2020, 13. 10.3390/en13061412

[32] Zhang M.; Cai Y.; Yang X.; Li L. Charging demand distribution analysis method of household electric vehicles considering users’ charging difference. *Electr*. *Power Autom*. *Equip*. 2020, 40, 154–161.

[33] Hafez O.; Bhattacharya K. Queuing Analysis Based PEV Load Modeling Considering Battery Charging Behavior and Their Impact on Distribution System Operation. *IEEE Trans*. *Smart Grid* 2018, 9, 261–273. 10.1109/tsg.2016.2550219

[34] Huang W.; Deng M.; Ge L.; He J.; He Z.; Luo J. Layout optimization strategy of hydrogen production and refueling stations considering the coupling effect of distribution network and hydrogen fuel vehicles. *High Volt*. *Eng*. 2022, *in press*.

[35] Long X.; Yang J.; Wu F.; Zhan X.; Lin Y.; Xu J. Prediction of Electric Vehicle Charging Load Considering Interaction Between Road Network and Power Grid and User’s Psychology. *Autom*. *Electr*. *Power Syst*. 2020, 44, 86–93.

[36] Quan H.F.; Li S.B.; Wei H.J.; Hu J.J. Personalized Product Evaluation Based on GRA-TOPSIS and Kansei Engineering. *Symmetry-Basel* 2019, 11, 21. 10.3390/sym11070867

[37] Zhang J.L.; Pei Y.Q.; Shen J.M.; Wang L.L.; Ding T.; Wang S. Charging Strategy Unifying Spatial-Temporal Coordination of Electric Vehicles. *IEEE Access* 2020, 8, 74853–74863. 10.1109/access.2020.2987607

[38] Shao Y.; Mu Y.; Lin J.; Wang K.; Gong Y. Fast Charging Guidance Strategy for Multiple Demands of Electric Vehicle, Fast Charging Station and Distribution Network. *Autom*. *Electr*. *Power Syst*. 2019, 43, 60–66 and 101.

[39] Fu Z.; Zhu W.; Zhu J.; Yuan Y. Fast charging guidance and pricing strategy for electric taxis based on dynamic traffic-grid coupling network. *Electr*. *Power Autom*. *Equip*. 2022, 42, 9–17.

