# Supplementary material for: Comprehensive evaluation of electric vehicle charging network under the coupling of traffic network and power grid
Source: PLoS One. 2022 Sep 23;17(9):e0275231. doi: 10.1371/journal.pone.0275231 (PMC9506653; doi:10.1371/journal.pone.0275231)
Supplement: S1 Appendix — (DOCX) [file pone.0275231.s001.docx]

**Appendix A**

**Table A1.** Road parameters and saturation

| **Road**  **section** | **Length**  **/km** | **Capacity** * | **7:00-9:00** | **9:00-12:00** | **12:00-14:00** | **14:00-17:00** | **17:00-19:00** | **19:00-23:00** | **Other**  **periods** |
| --- | --- | --- | --- | --- | --- | --- | --- | --- | --- |
| 1-2 | 6 | 3452 | 0.15 | 0.30 | 0.45 | 0.45 | 0.50 | 0.40 | 0.10 |
| 1-3 | 4 | 2566 | 0.10 | 0.25 | 0.35 | 0.40 | 0.40 | 0.40 | 0.20 |
| 2-6 | 5 | 3073 | 0.30 | 0.63 | 0.60 | 0.55 | 0.55 | 0.50 | 0.20 |
| 3-4 | 4 | 2655 | 0.37 | 0.45 | 0.50 | 0.56 | 0.60 | 0.55 | 0.20 |
| 3-12 | 4 | 2783 | 0.30 | 0.35 | 0.55 | 0.50 | 0.45 | 0.45 | 0.25 |
| 4-5 | 2 | 1428 | 0.40 | 0.56 | 0.60 | 0.55 | 0.60 | 0.50 | 0.30 |
| 4-11 | 6 | 3604 | 0.45 | 0.64 | 0.55 | 0.67 | 0.65 | 0.55 | 0.35 |
| 5-6 | 4 | 2433 | 0.35 | 0.42 | 0.45 | 0.50 | 0.45 | 0.38 | 0.20 |
| 5-9 | 5 | 2966 | 0.43 | 0.55 | 0.60 | 0.55 | 0.59 | 0.45 | 0.30 |
| 6-8 | 2 | 1569 | 0.22 | 0.35 | 0.43 | 0.40 | 0.40 | 0.35 | 0.10 |
| 7-8 | 5 | 3117 | 0.15 | 0.30 | 0.30 | 0.40 | 0.40 | 0.30 | 0.10 |
| 7-18 | 3 | 1589 | 0.15 | 0.31 | 0.35 | 0.35 | 0.40 | 0.40 | 0.10 |
| 8-9 | 10 | 4522 | 0.40 | 0.72 | 0.60 | 0.75 | 0.65 | 0.58 | 0.20 |
| 8-16 | 5 | 2877 | 0.35 | 0.46 | 0.45 | 0.50 | 0.75 | 0.45 | 0.20 |
| 9-10 | 3 | 1748 | 0.45 | 0.83 | 0.65 | 0.75 | 0.83 | 0.55 | 0.30 |
| 10-11 | 5 | 3056 | 0.40 | 0.65 | 0.70 | 0.65 | 0.80 | 0.50 | 0.20 |
| 10-15 | 6 | 3655 | 0.48 | 0.60 | 0.75 | 0.60 | 0.70 | 0.45 | 0.40 |
| 10-16 | 4 | 2649 | 0.45 | 0.70 | 0.70 | 0.55 | 0.65 | 0.40 | 0.40 |
| 10-17 | 8 | 3744 | 0.38 | 0.55 | 0.60 | 0.60 | 0.59 | 0.55 | 0.25 |
| 11-12 | 6 | 3389 | 0.30 | 0.42 | 0.45 | 0.50 | 0.65 | 0.40 | 0.20 |
| 11-14 | 4 | 2477 | 0.40 | 0.60 | 0.55 | 0.62 | 0.78 | 0.50 | 0.40 |
| 12-13 | 3 | 1664 | 0.20 | 0.35 | 0.40 | 0.35 | 0.40 | 0.30 | 0.20 |
| 13-24 | 4 | 2516 | 0.25 | 0.52 | 0.65 | 0.60 | 0.67 | 0.45 | 0.30 |
| 14-15 | 5 | 2976 | 0.45 | 0.63 | 0.65 | 0.50 | 0.72 | 0.55 | 0.20 |
| 14-23 | 4 | 2722 | 0.46 | 0.55 | 0.60 | 0.65 | 0.70 | 0.65 | 0.30 |
| 15-19 | 3 | 1722 | 0.40 | 0.65 | 0.59 | 0.55 | 0.65 | 0.60 | 0.40 |
| 15-22 | 3 | 1833 | 0.45 | 0.55 | 0.60 | 0.72 | 0.65 | 0.60 | 0.30 |
| 16-17 | 2 | 1576 | 0.50 | 0.45 | 0.55 | 0.50 | 0.63 | 0.55 | 0.40 |
| 16-18 | 3 | 1837 | 0.35 | 0.55 | 0.40 | 0.60 | 0.65 | 0.35 | 0.30 |
| 17-19 | 2 | 1653 | 0.40 | 0.65 | 0.55 | 0.55 | 0.75 | 0.50 | 0.40 |
| 18-20 | 4 | 2384 | 0.40 | 0.50 | 0.35 | 0.45 | 0.55 | 0.30 | 0.30 |
| 19-20 | 4 | 2245 | 0.30 | 0.25 | 0.30 | 0.25 | 0.30 | 0.40 | 0.20 |
| 20-21 | 6 | 3751 | 0.35 | 0.25 | 0.35 | 0.25 | 0.40 | 0.25 | 0.20 |
| 20-22 | 5 | 3261 | 0.35 | 0.30 | 0.45 | 0.35 | 0.40 | 0.40 | 0.30 |
| 21-22 | 2 | 1460 | 0.30 | 0.60 | 0.70 | 0.45 | 0.80 | 0.45 | 0.40 |
| 21-24 | 3 | 1649 | 0.40 | 0.40 | 0.40 | 0.40 | 0.50 | 0.30 | 0.40 |
| 22-23 | 4 | 2518 | 0.35 | 0.54 | 0.50 | 0.55 | 0.55 | 0.40 | 0.30 |
| 23-24 | 2 | 1534 | 0.40 | 0.40 | 0.80 | 0.40 | 0.35 | 0.40 | 0.40 |

* Maximum number of vehicles per road section.

**Table A2.** Parameter settings

| **Parameters** | **Value** | **Parameters** | **Value** |
| --- | --- | --- | --- |
| *v_ij,0_* | 60km/h | *ϑ* | 0.5 |
| *φ* | 1.726 | *r_0_* | 0.08 |
| γ | 3.15 | *k_ser_* | 0.03 |
| *P_cool_* | 1.2kW | *z* | 15 |
| *P_heat_* | 1.5kW | *M_car_* | 240gCO_2_/km |
| *Cap_r_* | 55kW∙h | *η_grid_* | 94.4% |
| *P_c_* | 60kW | *k_1_* | 0.45 |
| *η_c_* | 85% | *k_2_* | 0.55 |

**Table A3.** Nodal correspondence between traffic network and distribution network

| **Road network node** | **Power grid node** | **Road network node** | **Power grid node** |
| --- | --- | --- | --- |
| 1 | 1 | 13 | 20 |
| 2 | 25 | 14 | 5 |
| 3 | 2 | 15 | 6 |
| 4 | 3 | 16 | 15 |
| 5 | 23 | 17 | 14 |
| 6 | 24 | 18 | 30 |
| 7 | 29 | 19 | 13 |
| 8 | 28 | 20 | 31 |
| 9 | 27 | 21 | 10 |
| 10 | 26 | 22 | 11 |
| 11 | 4 | 23 | 8 |
| 12 | 19 | 24 | 9 |

**Table A4.** Initial index value

| **Criterion layer** | **Index layer** | **Scheme 1** | **Scheme 2** | **Scheme 3** | **Scheme 4** |
| --- | --- | --- | --- | --- | --- |
| User feedback | EV demand satisfaction | 3.95 | 3.87 | 3.74 | 3.81 |
|  | Average charging time | 0.684 | 0.657 | 0.692 | 0.713 |
|  | EV charging cost | 35.42 | 34.71 | 37.19 | 36.67 |
| Charging network  operation situation | Charging station utilization rate | 0.63 | 0.71 | 0.68 | 0.75 |
|  | Scope of services | 1.73 | 2.01 | 0.83 | 1.87 |
|  | Annual construction and operating costs | 429.86 | 408.51 | 457.34 | 474.18 |
| Influence of road  network operation | Road network vulnerability | 1.84 | 1.92 | 2.17 | 1.77 |
|  | Carbon emission reduction benefits | 1274.21 | 1194.65 | 1428.54 | 1175.32 |
|  | Change amount of traffic  network operation index | 107.23 | 110.48 | 112.74 | 108.17 |
| Influence of power  grid operation | Network loss | 0.985 | 1.207 | 0.926 | 0.819 |
|  | Voltage deviation non-out of limit rate | 0.837 | 0.881 | 0.875 | 0.894 |
|  | Network security operation index | 0.012 | 0 | 0.024 | 0 |
|  | Variation of load peak-valley difference | 8.86 | 8.12 | 8.52 | 8.24 |

**Table A5.** Indicator standardized data

| **Criterion layer** | **Index layer** | **Scheme 1** | **Scheme 2** | **Scheme 3** | **Scheme 4** |
| --- | --- | --- | --- | --- | --- |
| User feedback | EV demand satisfaction | 1 | 0.619 | 0 | 0.333 |
|  | Average charging time | 0.518 | 1 | 0.375 | 0 |
|  | EV charging cost | 0.714 | 1 | 0 | 0.210 |
| Charging network  operation situation | Charging station utilization rate | 0 | 0.667 | 0.417 | 1 |
|  | Scope of services | 0.237 | 0 | 1 | 0.119 |
|  | Annual construction and operating costs | 0.675 | 1 | 0.256 | 0 |
| Influence of road  network operation | Road network vulnerability | 0.825 | 0.625 | 0 | 1 |
|  | Carbon emission reduction benefits | 0.391 | 0.076 | 1 | 0 |
|  | Change amount of traffic  network operation index | 1 | 0.419 | 0 | 0.846 |
| Influence of power  grid operation | Network loss | 0.572 | 0 | 0.724 | 1 |
|  | Voltage deviation non-out of limit rate | 0 | 0.772 | 0.667 | 1 |
|  | Network security operation index | 0.5 | 1 | 0 | 1 |
|  | Variation of load peak-valley difference | 0 | 1 | 0.459 | 0.838 |

**Table A6.** Euclidean distance and gray correlation under different schemes

|  | **Metric** | **Scheme 1** | **Scheme 2** | **Scheme 3** | **Scheme 4** |
| --- | --- | --- | --- | --- | --- |
| User feedback | $D_{i}^{+}$ | 0.429 | 0.421 | 0.321 | 0.332 |
|  | $D_{i}^{-}$ | 0.266 | 0.332 | 0.482 | 0.343 |
|  | $V_{i,j}^{+}$ | 0.480 | 0.575 | 0.778 | 0.588 |
|  | $V_{i,j}^{-}$ | 0.665 | 0.674 | 0.556 | 0.519 |
| Charging network  operation situation | $D_{i}^{+}$ | 0.445 | 0.389 | 0.381 | 0.374 |
|  | $D_{i}^{-}$ | 0.306 | 0.382 | 0.276 | 0.389 |
|  | $V_{i,j}^{+}$ | 0.572 | 0.644 | 0.517 | 0.584 |
|  | $V_{i,j}^{-}$ | 0.682 | 0.587 | 0.571 | 0.651 |
| Influence of road  network operation | $D_{i}^{+}$ | 0.409 | 0.362 | 0.311 | 0.389 |
|  | $D_{i}^{-}$ | 0.334 | 0.359 | 0.442 | 0.338 |
|  | $V_{i,j}^{+}$ | 0.568 | 0.622 | 0.650 | 0.601 |
|  | $V_{i,j}^{-}$ | 0.617 | 0.541 | 0.584 | 0.622 |
| Influence of power  grid operation | $D_{i}^{+}$ | 0.019 | 0.013 | 0.012 | 0.010 |
|  | $D_{i}^{-}$ | 0.001 | 0.007 | 0.018 | 0.013 |
|  | $V_{i,j}^{+}$ | 0.447 | 0.6 | 0.621 | 0.685 |
|  | $V_{i,j}^{-}$ | 0.637 | 0.575 | 0.673 | 0.579 |
